# Aerodynamic Drag Reduction in Commercial Vehicle Using CFD-Based Design Optimisation

**DOI:** 10.12688/f1000research.163917.1

**Published:** 2025-08-26

**Authors:** Madhav S Prabhu, Sudheendra Prabhu K, Amar A Murthy, Srinivas G

**Affiliations:** 1 Department of Aeronautical & Automobile Engineering, Manipal Institute of Technology (MIT), Manipal Academy of Higher Education (MAHE), Manipal, Udupi, Karnataka, 576104, India; 2 Department of Mechanical & Industrial Engineering, Manipal Institute of Technology (MIT), Manipal Academy of Higher Education (MAHE), Manipal, Udupi, Karnataka, 576104, India

**Keywords:** Truck, CFD, Aerodynamics, Drag, Optimization, Flow control techniques

## Abstract

**Background:**

The aerodynamics of commercial vehicles is complex. The bluff body of a truck creates complex airflow patterns and induces drag. Numerous researchers have concentrated on enhancing the design of passenger cars, whereas the aerodynamic design of trucks has been largely overlooked. Truck operators are often unaware of the drag force acting on the truck and its impact on annual fuel consumption. Trucks can save large amounts of fuel annually by improving their aerodynamics. Most trucks are currently operating on the road without any frontal wind deflector, which causes drag accumulation over the frontal region of the truck.

**Method:**

This study aims to reduce the drag force of an SCV through effective design optimization using the ANSYS Fluent tool. Using CFD methods and various numerical techniques, a comprehensive 3D airflow analysis was conducted on a scaled-down truck model. Subsequently, design optimizations were performed on the baseline truck to enhance its aerodynamic efficiency. A systematic literature review was conducted to obtain insights into the flow field of commercial vehicle drag-reduction techniques.

**Results:**

Flow analysis was performed using different turbulence models, and the results were validated using available literature data. To improve the aerodynamic performance of the truck, different geometrical optimization models were tested on an iterative basis. Each model was further compared with the baseline model to obtain more reliable results. An airflow analysis was conducted for different air velocities. The obtained drag values for both the aerodynamically optimized truck and the baseline truck were compared and analyzed. Effective design alterations led to an 18% reduction in drag.

**Conclusions:**

The enhanced aerodynamic outcomes are presented in detail for the optimized truck model. This study will help automobile engineers and truck researchers to understand the flow aerodynamics of commercial vehicles and improve their aerodynamic efficiency.

**List of abbreviations**
CFD: Computational Fluid Dynamics
CNG: Compressed Natural Gas
CO _2_
: Carbon Dioxide
CRF: Cab Roof Fairing
DDES: Delayed Detached Eddy Simulation
DES: Detached Eddy Simulation
LCV: Large Commercial Vehicle
LES: Large Eddy Simulation
LPG: Liquified Petroleum Gas
PIV: Particle Image Velocimetry
RANS: Reynolds Averaged Navier Stokes
SCV: Small Commercial Vehicle
SST: Shear Stress Transport
SUV: Sports Utility Vehicle
VG: Vortex Generator
WT: Wind Tunnel

**List of symbols**
C _D_
: Drag coefficient
F _D_
: Drag force
H: Height
W: Width
L: Length
G: Gap
V: Velocity
A: Area
Θ: Yaw angle
β: Flap slant angle
α: Frontal deflector angle
ρ: Air density
R _e_
: Reynolds number

## 1. Introduction

Aerodynamics in automobile vehicle design plays a very important role, as they directly impact a vehicle's fuel efficiency and overall performance. By reducing the air resistance, vehicles can improve their fuel economy. An improved aerodynamic design reduces wind noise at higher speeds and enhances vehicle handling. Drag is classified into three main types: skin friction drag, induced drag, and form drag. Owing to the boxy shape of heavy commercial vehicles, truck vehicles experience more air resistance. A drag is a prominent force acting on a vehicle at higher speeds. Most trucks are not aerodynamically efficient, and by optimizing truck design, the fuel consumption can be reduced by up to 20%. ^1^ Modern automotive designs focus on vehicle frontal wind deflector designs and the streamlined shape of vehicles. An optimal design ensures smooth airflow around the vehicle body and reduces the overall drag force.

Commercial trucks are the backbone of the transport industry and play an essential role in supplying goods over long distances, in supply chains, and in logistics. The main advantages of commercial vehicles in the transport industry include their high load-carrying capacity and durability. Limited maneuverability in cities and other environmental concerns, such as high carbon emissions and high fuel consumption, are some limitations. The importance of aerodynamic design is a major topic for discussion. Drag arises mainly from vehicle shape **,** installing aerodynamic devices such as cab-side extenders and frontal wind deflectors will improve fuel economy and lower carbon emissions.

**Figure 1.**
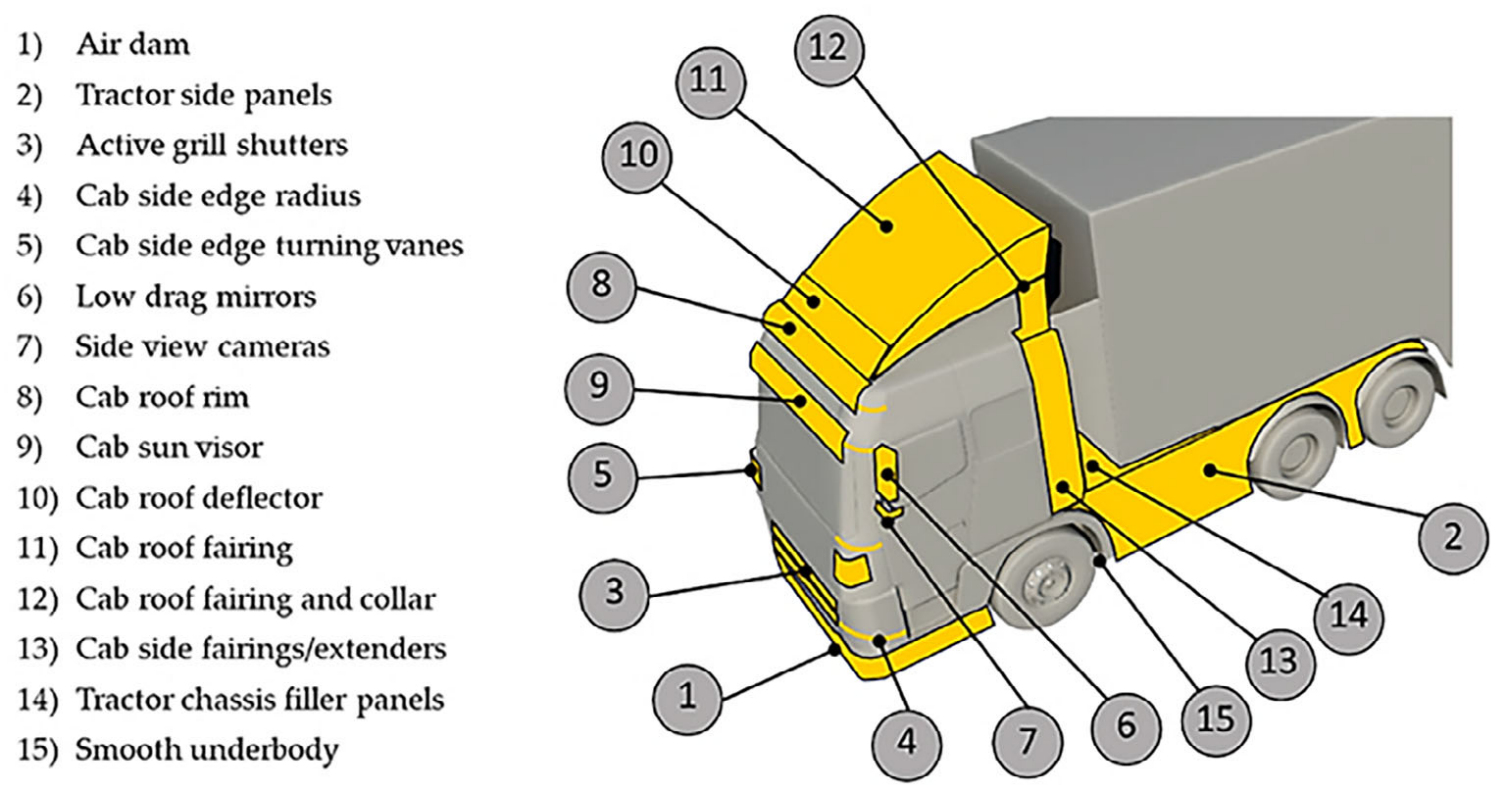
Various aerodynamic devices installed on a heavy commercial vehicle. ^1^

Figure 1 shows recent developments in truck design. Streamlining the truck body with various devices and a sleeker shape to reduce the resistance is the most common and effective method. Aerodynamic enhancements such as cab roof fairing and gap enclosures help minimize vehicle drag at highway speeds. Aerodynamic enhancements such as cab roof fairing and gap enclosures help minimize vehicle drag at highway speeds. Electric vehicles in the commercial category have also been developed and are emerging in many European countries. In terms of safety, top-model commercial vehicles in many European countries also include features such as cruise control and advanced lane-keeping assistance systems. Additional aerodynamic features, such as active grille shutters, are used for cooling engine components whenever needed. Overall, these innovations aim to reduce drag and improve truck performance, which helps the trucking industry reduce greenhouse gas emissions and align with global environmental sustainability goals.

## 2. Literature review

A systematic literature review was conducted in the field of vehicle drag reduction using wind-tunnel methods. ^2– 4^ In summary, passive drag reduction strategies have shown significant potential for reducing aerodynamic drag. Simplified bluff bodies, such as the Ahmed body, are the most widely used vehicle models for research purposes, ^5^ and have been used as benchmarks for different numerical and experimental studies. Techniques such as vortex generators, ^6^ rear cavities, and devices such as fences or forward-facing panels can effectively reduce drag by controlling the airflow and delaying separation. For instance, small-scale wind tunnel tests have reported drag reductions exceeding 50% using drag-reduction methods. However, the drag reduction results from simplified models must be approached cautiously. ^7, 8^ Simplified bodies can only offer approximate indicators of real-world performance. Future research must consistently verify the effects of drag reduction devices on fully detailed vehicles in actual driving scenarios. ^9^

Similarly, a review of the literature on numerical methods was performed, focusing on various turbulence models. The use of diverse numerical techniques for analyzing the flow remains essential for understanding the flow characteristics of a vehicle. The comparison of drag suggests that realizable K-epsilon provides greater accuracy than DDES. ^10^ LES is a reliable method for studying various aerodynamic loads and is highly effective under different conditions. To analyze the flow around the truck model, utilizing turbulence models such as the realizable k-ε and k-omega SST in the ANSYS solver will yield more accurate outcomes. ^11^ Implementing these models will significantly decrease the computational cost, with the realizable k–ε model giving the closest value to the experimental data and with the least error. Hybrid RANS/LES models have proven to be more effective than the RANS turbulence model in predicting the flow over vehicles, while still maintaining reasonable computational costs. ^12, 13^ Comparisons of the drag indicate that the realizable k–ε model is more precise than the SST and DDES/SST models. The utilization of the DNS method in captures all turbulence scales, giving highly accurate results, although it incurs high computational costs. ^14^ The selection of DNS, RANS, and LES techniques depends on the specific research type, available computational resources, and the desired level of result accuracy.

### 2.1 Research gaps

There are some limitations in the current research on 3D flow simulations for commercial trucks because of their complex body shapes. Because of the complex airflow patterns, greater computational resources are required to analyze the flow behavior. Although simplified models have been used to study small-scale vortices and turbulent wakes, full-scale simulations are crucial for understanding airflow interactions, especially under varying speeds. Underbody flow, including interactions between the rotating wheels and the ground, remains underexplored. Additionally, research on grid resolution, prism layer configurations, and turbulence models for commercial vehicles is insufficient compared to that for passenger cars. ^15^ Flow separation points, particularly at the rear of the truck, produce complex wake patterns that are often oversimplified in various studies. Key aerodynamic factors such as temperature, density, velocity, and turbulence have also not been thoroughly analyzed. ^16^ Addressing these gaps through full 3D simulations and improved numerical methods is essential for optimizing truck aerodynamics.

### 2.2 Research objectives

The objectives of the research are:

• To conduct an aerodynamic flow analysis of the truck and validate the results with experimental and numerical literature data.
• To investigate the aerodynamic flow performance parameters of a truck vehicle by incorporating various numerical techniques.

## 3. Methodology

**Figure 2.**
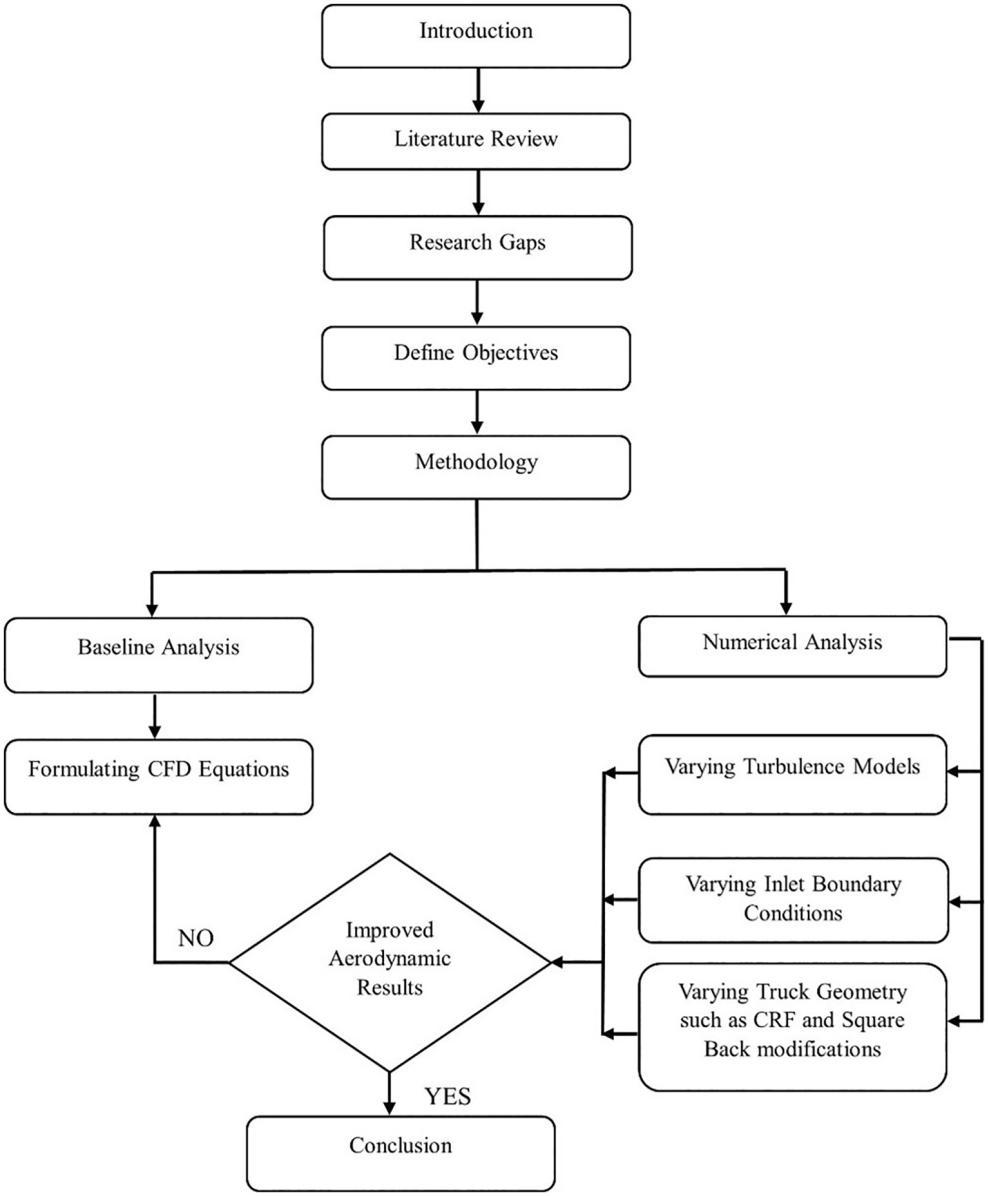
Methodology flowchart.

Figure 2 shows the flowchart of the study. The process begins with an introduction to vehicle aerodynamics and a comprehensive literature review on drag reduction methods, in which existing studies are explored to gather important insights. A summary of the literature review helps identify gaps in the current research field. After defining the objectives, a baseline analysis was performed, and the truck model used for the analysis was a Bedford J-series light commercial vehicle. ^17^ The computational fluid dynamics method is used, where numerical analysis focuses on various factors that affect aerodynamic behavior. Varying the turbulence models, inlet boundary conditions, and geometrical features of the vehicle helps to understand how different conditions and design modifications impact the overall flow field and drag reduction. Once the simulations were conducted, the results were evaluated to determine whether the required level of accuracy was obtained. If not, the truck design will be redesigned, and the final optimized model and simulation results are presented in this paper.

### 3.1 Baseline analysis

**3.1.1 Modelling**

In this study, CFD analysis was performed on a Bedford J Series truck model, chosen because of the easy availability of its blueprints, which facilitated the creation of a 3D model. In addition, this truck model is widely used in small commercial vehicle segments and is available in almost all countries. An experimental study was conducted using a similar model that focuses on drag reduction. Hence, the experimental data are publicly available to verify the accuracy of the CFD results.

The baseline model was a 1/10 ^th^ scaled-down model of the actual truck dimensions. Reference paper ^18^ experiments on similar scaled-down models. Hence, in this work, similar dimensions were used for modeling the truck using ANSYS DesignModeler, and the experimental data were publicly available to check the accuracy of the CFD results. The baseline dimensions of the truck geometry are presented in Table 1.

**Table 1.** Truck model dimensions (1/10 ^th^ scale). ^17^

Length (L)	0.57 m
Height (H)	0.25 m
Width (W)	0.22 m
Wheelbase (WB)	0.37 m
Truck Cabin-Trailer Gap (G)	0.02 m
Ground Clearance (GC)	0.035 m

**Figure 3.**
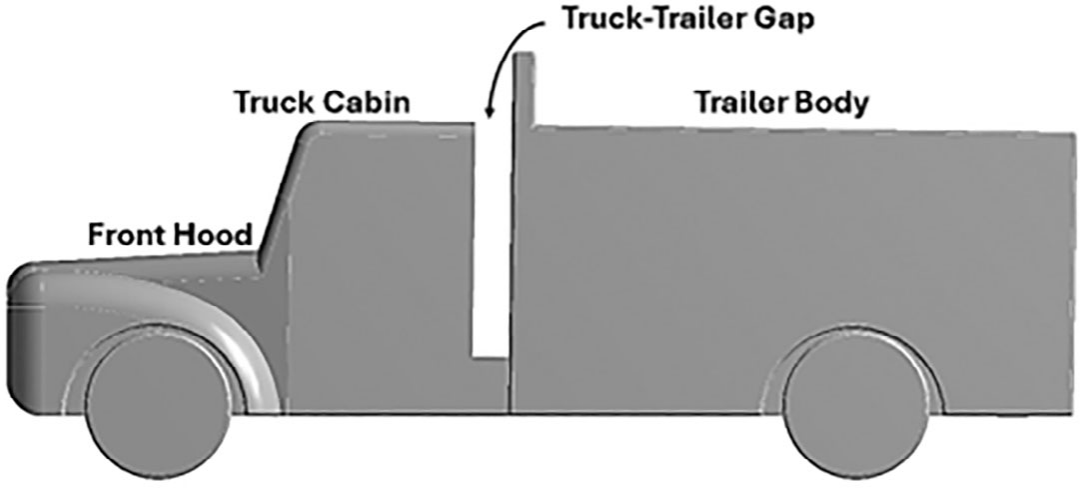
Bedford truck 3D model (Side view).

**Figure 4.**
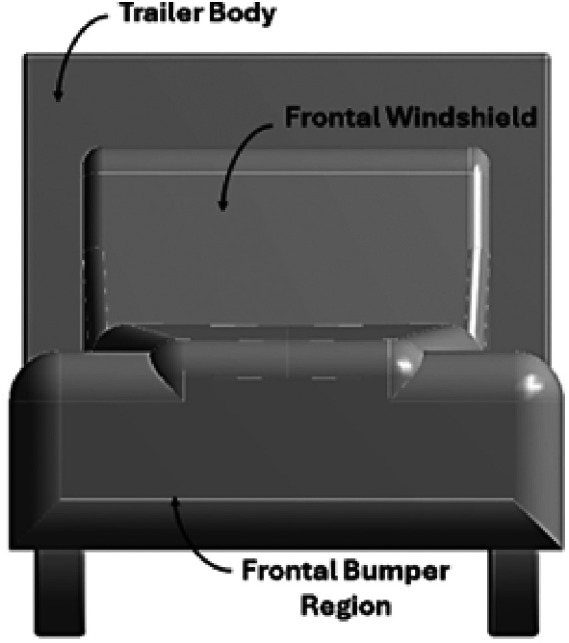
Bedford truck 3D model (Front view).

Figures 3 and 4 show the 3D models of the scaled-down Bedford J-series truck. The ANSYS DesignModeler and SpaceClaim tools have been extensively used for 3D modeling.

**Figure 5.**
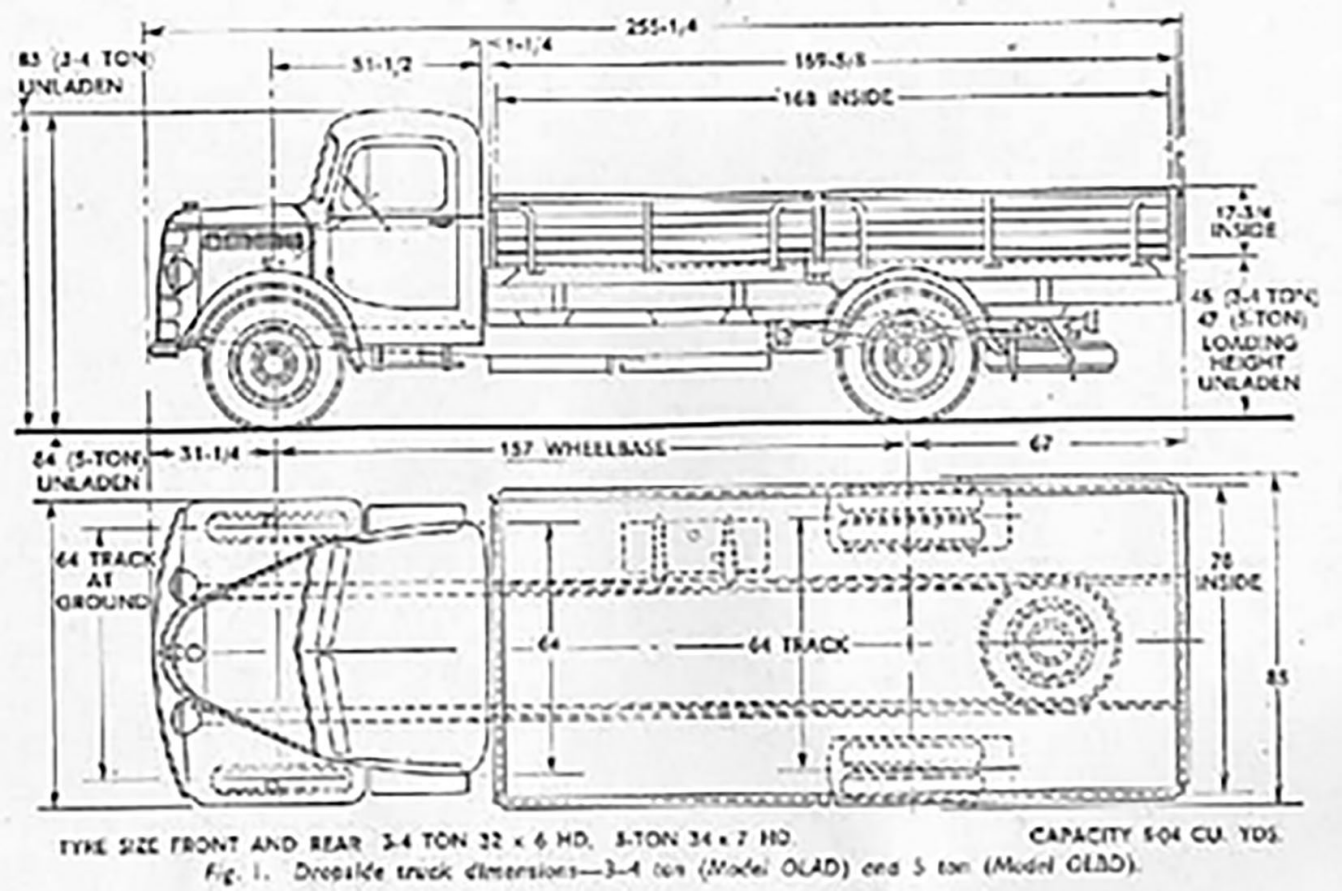
Blueprint of the Bedford J series truck. ^17^

Figure 5 shows blueprints of the Bedford truck. The dimensions of the reference paper were the same as the actual size. For this simulation, the actual dimensions were scaled to a factor of 10.

**3.1.2 Meshing**

The mesh was generated using ANSYS meshing module. For the initial meshing, a tetrahedral mesh was implemented, consisting of 6.2 million elements and 1.8 million nodes. An unstructured tetrahedral mesh is typically used for analyzing turbulent flows. Regarding the mesh quality, the average element quality was 0.73, while the maximum skewness value was 0.92. An orthogonal quality of 0.99 was obtained, with an average of 0.76. The named selections, surface mesh size, and other mesh details are listed in Table 2.

**Table 2.** Mesh details for the truck and enclosure.

Geometry	Selection type	Mesh details
Outer Domain	Body	18.5 – 21.5 mm
Truck Surface	Named Selection – Face Sizing	1.5 – 3 mm
Truck Surface	Named Selection – Inflation First Layer Thickness	0.35 mm
	Number of Layers (N)	5
	Growth Rate	1.2

**Figure 6.**
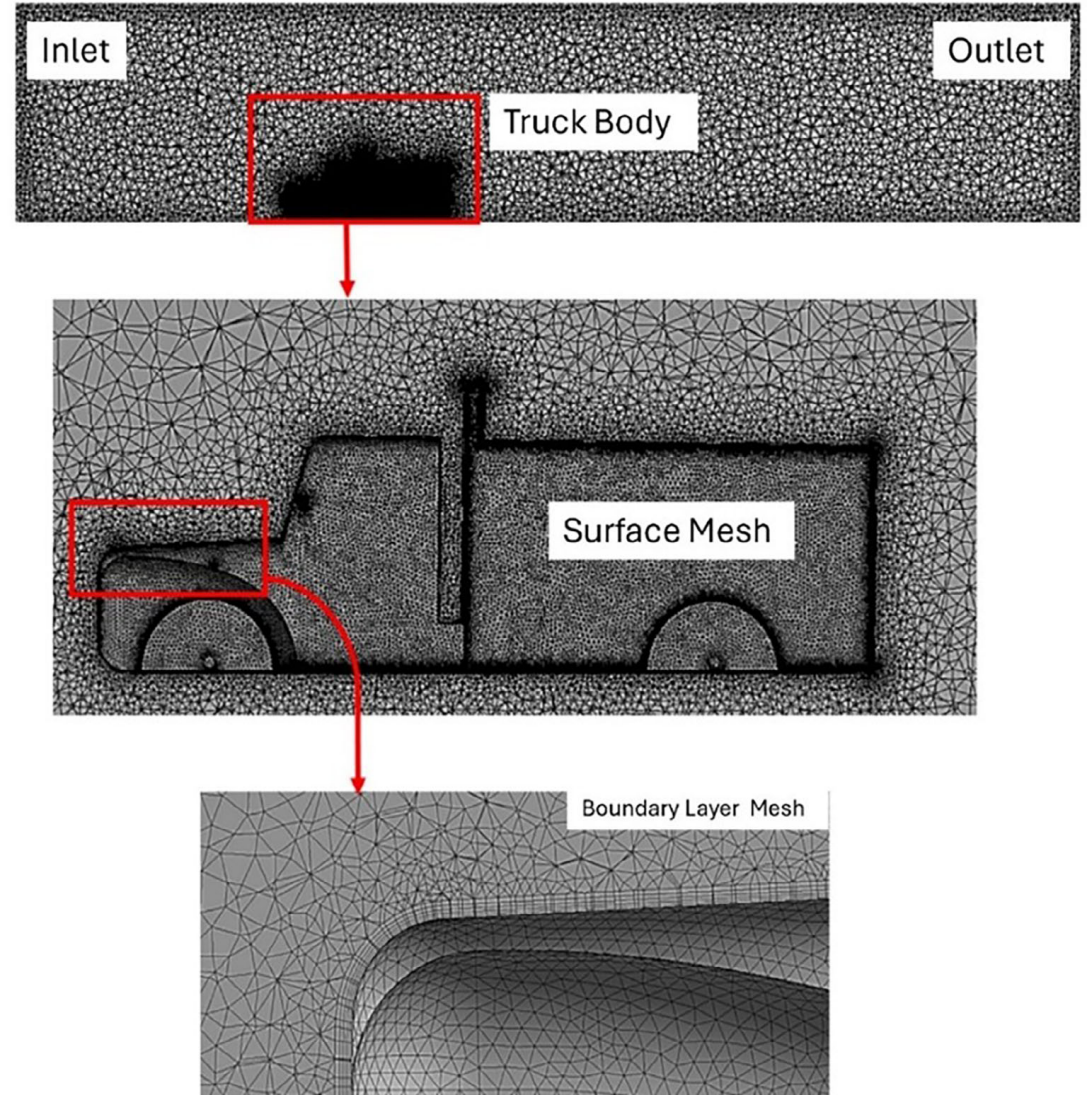
Mesh generated for the enclosure and the truck surface.

Figure 6 shows the complete mesh generated in the ANSYS mesh module. The mesh size was set to 20 mm, whereas for the truck surface, it was set to 3 mm. The growth rate for the inflation layer was set to a default of 1.2, and the first layer thickness height was set to 0.3 mm with a maximum number of five layers to capture the boundary layer.

**3.1.3 Boundary condition**

**Figure 7.**
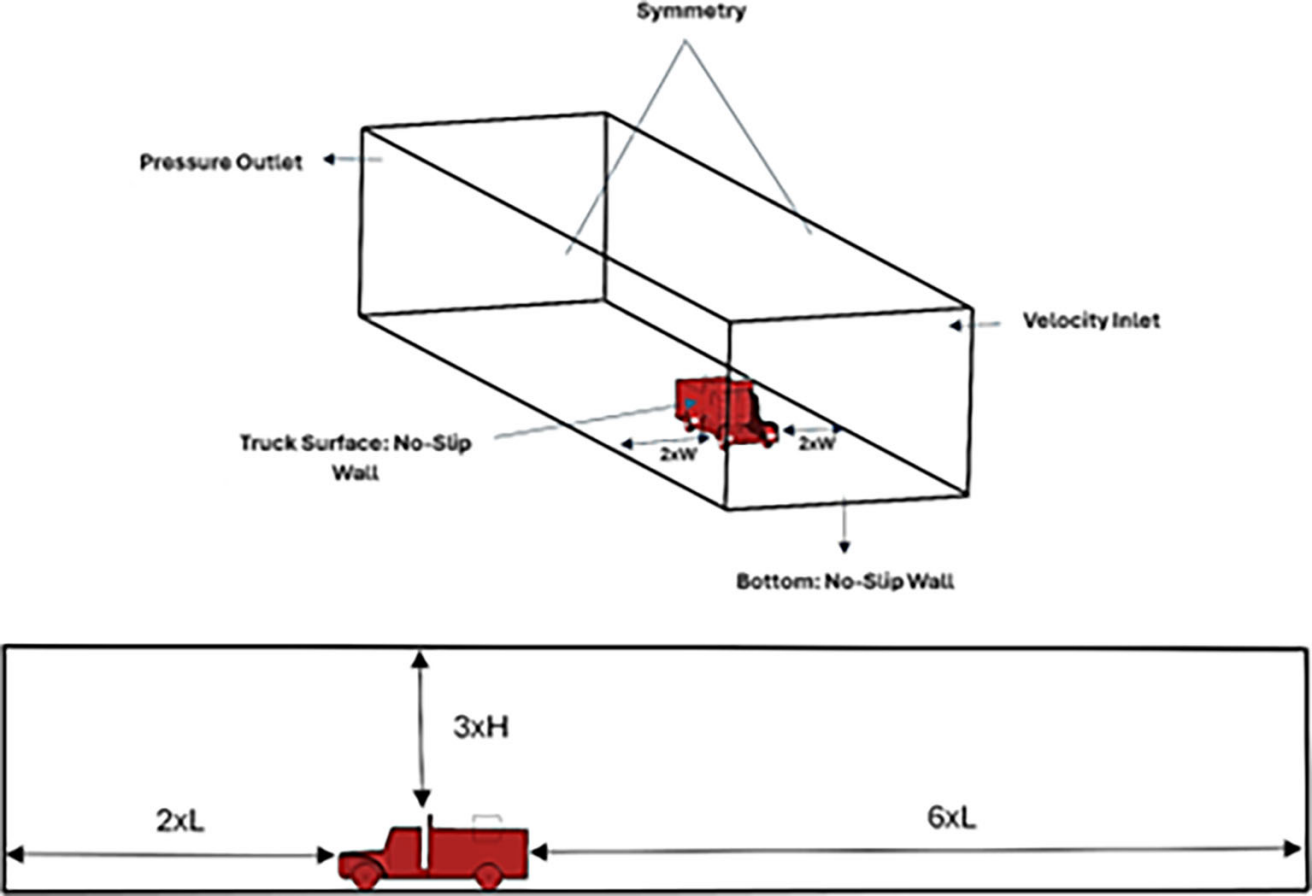
Computational fluid domain dimensions and boundary conditions.

After modeling the truck, a computational fluid domain that encompasses the truck model was established to simulate the airflow around the truck. The volume was established by subtracting the truck body using the Boolean function in DesignModeler. The truck length was six times the rear wall, two times the truck length to the front wall, two times the width of the truck to the side walls, and three times the truck height to the top wall. The boundary conditions used for the domain are shown in Figure 7, and the boundary conditions are listed in Table 3.

**Table 3.** Boundary conditions for the named selections.

Named selection	Boundary type
Inlet	Velocity -Inlet
Outlet	Pressure -Outlet
Exterior Walls	Symmetry
Road	No-Slip Wall condition
Truck Surface	No-Slip Wall condition

**Figure 8.**
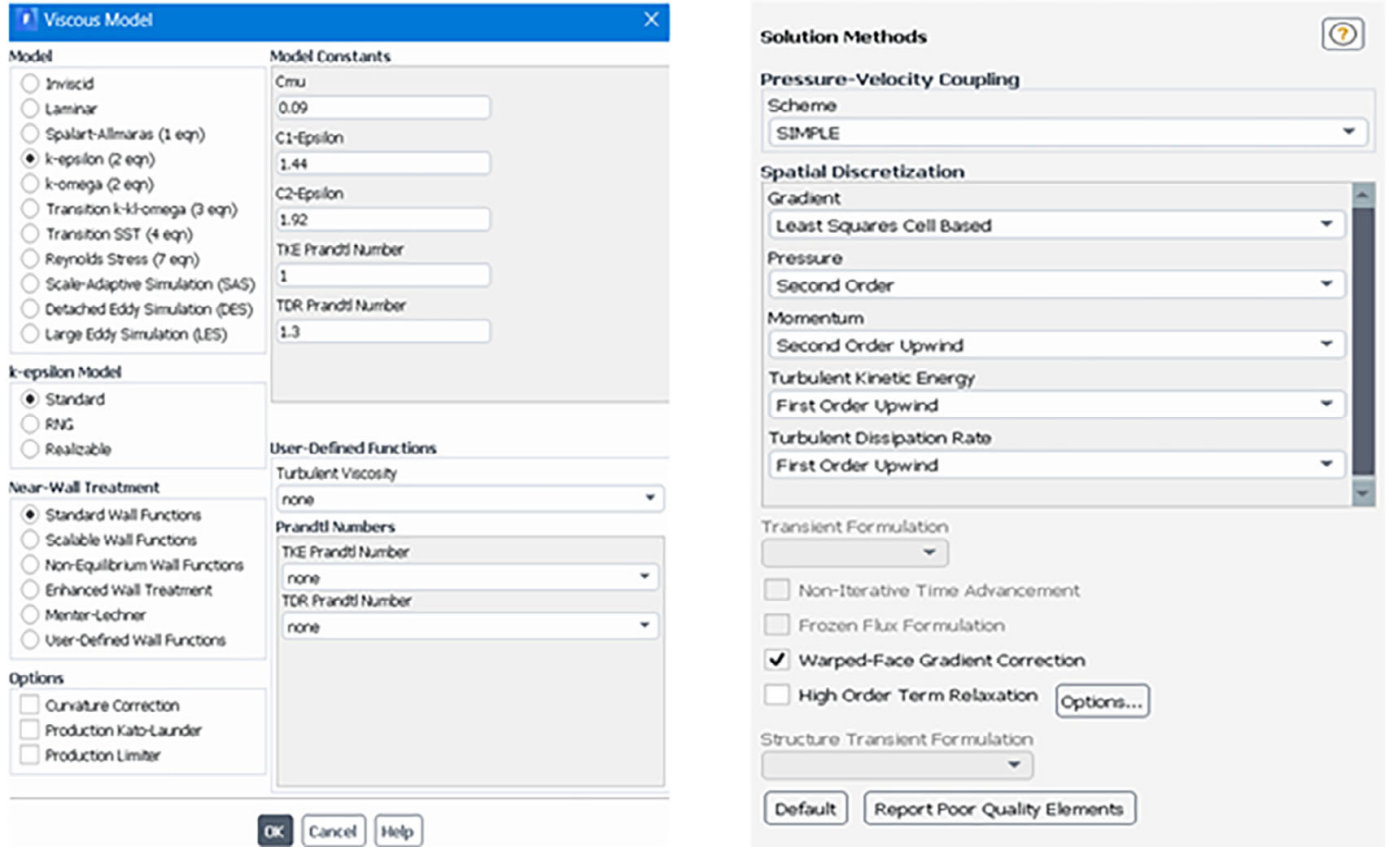
Turbulence model settings and solution methods.

The solver was set up according to the model type and operating conditions. Operating conditions, such as atmospheric pressure for the truck at sea level, were assumed. The solver uses a pressure-based incompressible steady-state flow condition. Figure 8 shows the settings for the turbulence model, where the K-Epsilon (2 Equation) model was used, and the fluid properties of air were constant with a density of 1.225 kg/m ^3^ ( Table 4).

**Table 4.** Results of grid independence test.

Mesh size (mm)	Number of elements (million)	Drag coefficient (C _D_)
21.5	6.01	0.476
21	6.11	0.533
20	6.23	0.547
19	6.38	0.551
18.5	6.47	0.554

**Figure 9.**
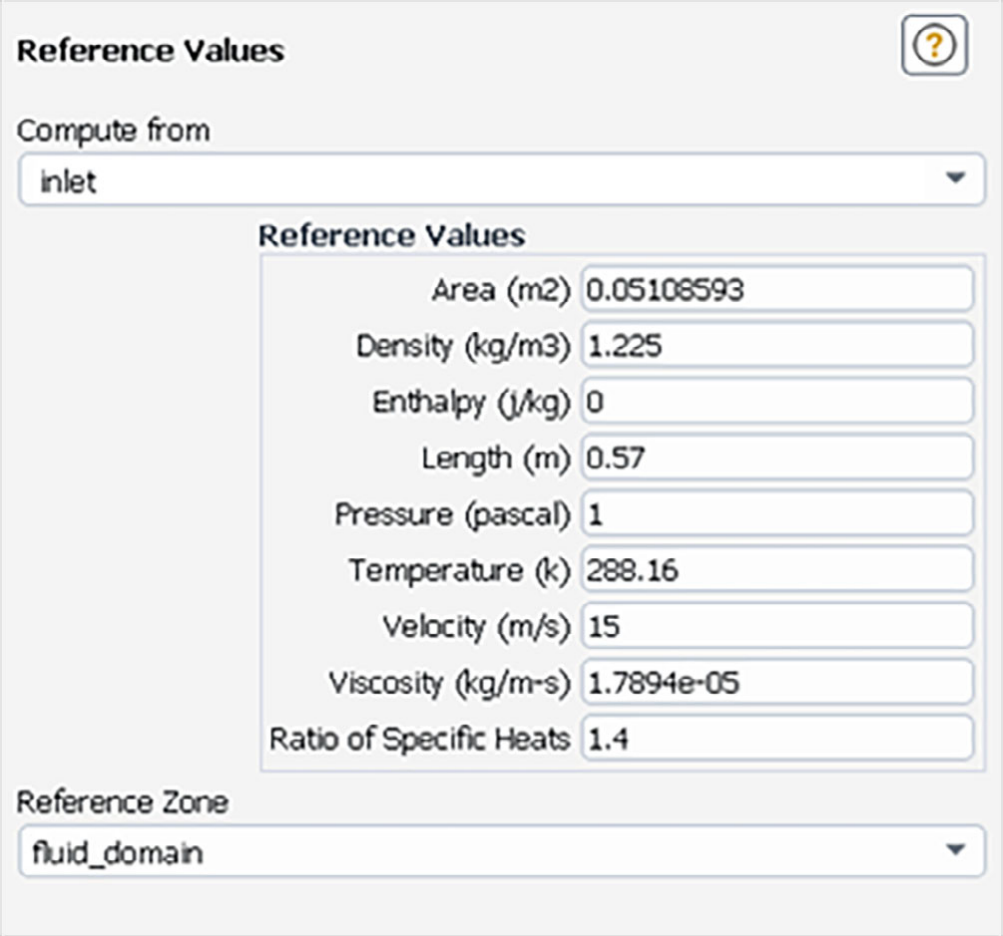
Reference values.

The Standard K-Epsilon model with standard wall functions was used, and the semi-implicit method for pressure-linked equations (SIMPLE) algorithm was used as the pressure-velocity coupling scheme. The details of the solution method are shown in Figure 8 and Figure 9 shows the reference values used for the solution. The reference area was calculated for the projected frontal area of the truck, and the reference length was scaled down by the truck length.

**3.1.4 Governing equations**

Fluent software solves these Navier-Stokes equations, composed of the continuity equation and momentum equations, as given below (1) – (4). ^19^

Continuity equation:

$$\frac{\mathrm{\partial u}}{\mathrm{\partial x}}+\frac{\mathrm{\partial v}}{\mathrm{\partial y}}+\frac{\mathrm{\partial w}}{\mathrm{\partial z}}=0$$ (1)

X-Momentum equation:

$$\rho \;\frac{\mathrm{Du}}{\mathrm{Dt}}=-\frac{\mathrm{\partial p}}{\partial x}+\mu \left(\frac{\partial^{2}u}{\partial x^{2}}+\frac{\partial^{2}u}{\partial y^{2}}+\frac{\partial^{2}u}{\partial z^{2}}\right)+\rho .f_{x}\;$$ (2)

Y-Momentum equation:

$$\rho \;\frac{\mathrm{Dv}}{\mathrm{Dt}}=-\frac{\mathrm{\partial p}}{\partial y}+\mu \left(\frac{\partial^{2}v}{\partial x^{2}}+\frac{\partial^{2}v}{\partial y^{2}}+\frac{\partial^{2}v}{\partial z^{2}}\right)+\rho .f_{y}\;$$ (3)

Z-Momentum equation:

$$\rho \;\frac{\mathrm{Dw}}{\mathrm{Dt}}=-\frac{\mathrm{\partial p}}{\partial z}+\mu \left(\frac{\partial^{2}w}{\partial x^{2}}+\frac{\partial^{2}w}{\partial y^{2}}+\frac{\partial^{2}w}{\partial z^{2}}\right)+\rho .f_{z}\;$$ (4)

Reynolds equation:

$$R_{e}=\frac{\rho .v.L}{\mu }$$ (5)

The Reynolds number is a dimensionless value that determines whether the fluid flow is laminar or turbulent or not. Equation (5) represents the Reynolds number, where ρ is the fluid density (kg/m ^3^), v is the fluid velocity (m/s), L is the characteristic length of the object (m), and μ is the dynamic viscosity of the fluid (kg/ms). The drag coefficient is represented in the simulations as a dimensionless value, illustrating the aerodynamic characteristics of the truck based on Equation (6). ^20^

$$C_{D}=\frac{F_{D}}{0.5\;\rho \;A\;v^{2}\;}$$ (6)

where C _D_ = Drag coefficient, F _D_ = Drag force (N), ρ is the fluid density (kg/m ^3^), A = Area (m ^2^), and v = Velocity (m/s).

**3.1.5 Drag coefficient plot**

**Figure 10.**
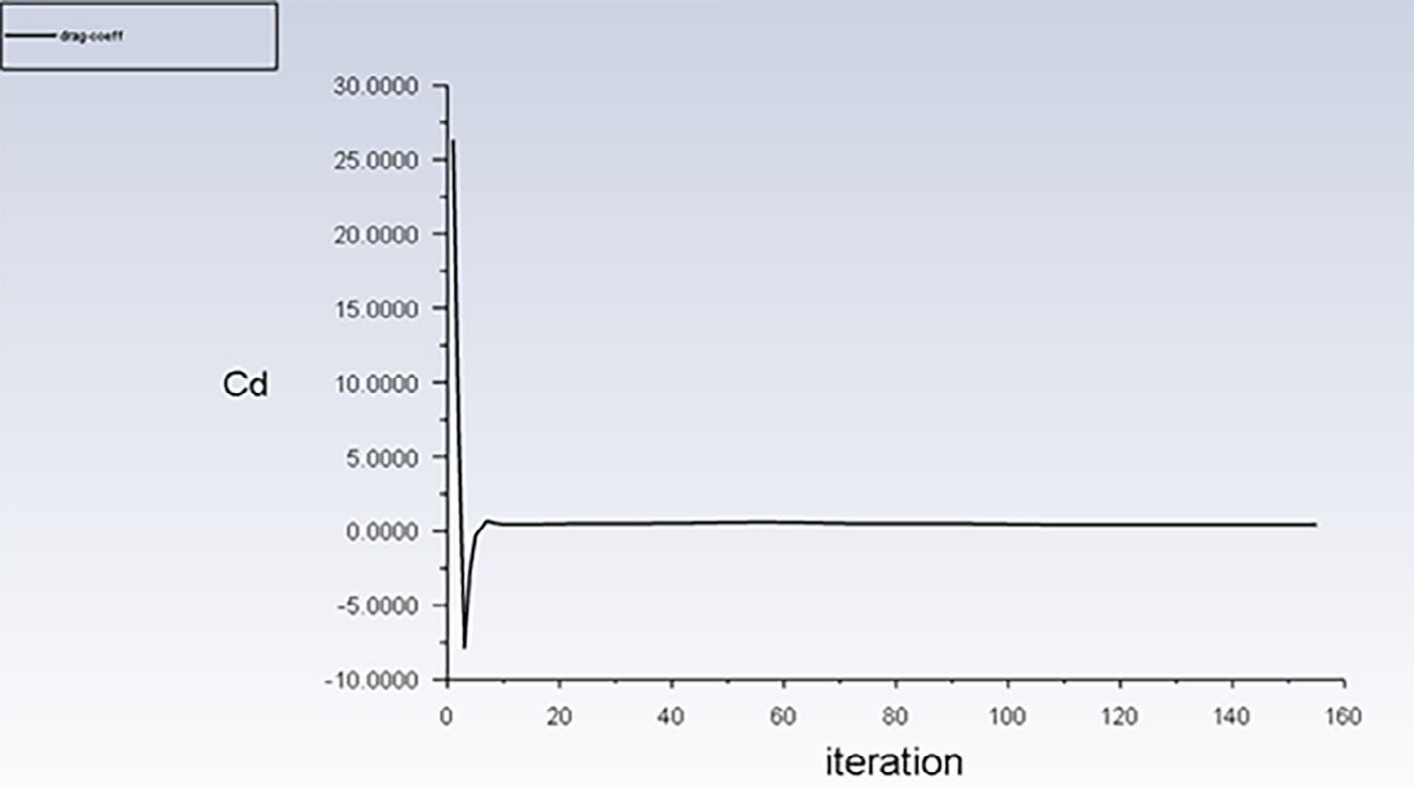
Drag coefficient plot for baseline model.

The simulation used the first-order upwind turbulent kinetic energy method. The report definition was used to calculate the drag coefficient (C _D_) for the truck surface. Figure 10 shows the C _D_ plot of the simulation results.

## 4. Results and discussions

A grid independence test was performed for baseline model analysis to ensure an optimal mesh. Various turbulence models were tested to determine the best grid size, and the obtained results were analyzed. A varying velocity test was performed for the best turbulence model, and the effect of varying the velocity on the drag coefficient value was observed and studied.

### 4.1 Grid independence test

A grid independence test was conducted to ensure that the obtained drag coefficient results did not depend on the grid used in the simulation. Five different mesh sizes were chosen: 6.0, 6.1, 6.2, 6.3 and 6.4 million mesh elements. The five simulation results showed that the mesh sizes of 6.2, 6.3, 6.4 million elements had the same drag value of C _D_ = 0.55. Hence, a 20 mm mesh size with 6.2 million elements was chosen for further simulation. The error percentages for 6.2, 6.3, 6.4 million elements were similar. This ensured an optimal mesh was used for further simulations.

**Figure 11.**
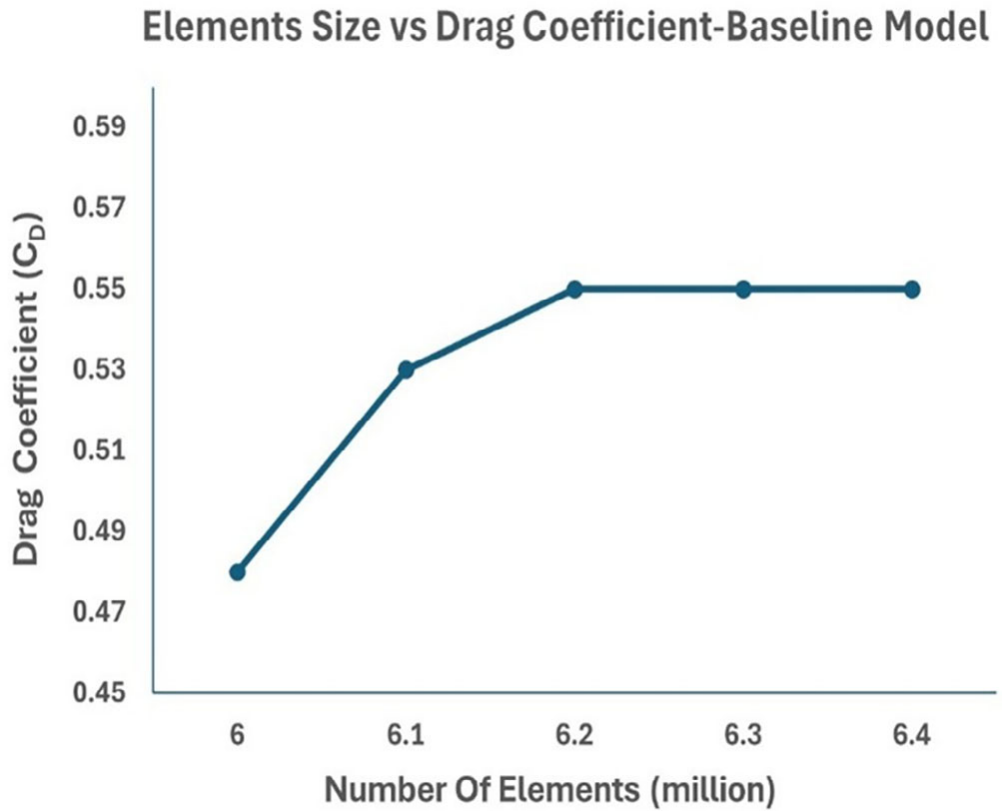
Number of elements vs drag coefficient for baseline model.

From Figure 11, it can be observed that the drag coefficient value remains constant at 6.2, 6.3 and 6.4 million elements. This ensured an optimal mesh was used for further simulations. The baseline mesh was set to 20 mm with 6.2 million mesh elements. The percentage error was obtained for the grid analysis.

### 4.2 Varying turbulence models

The model comparison results provided the best viscous model for further simulation, ensuring an accurate result with the least computational error. The drag coefficient value was compared with that in the reference paper. ^18^

**Figure 12.**
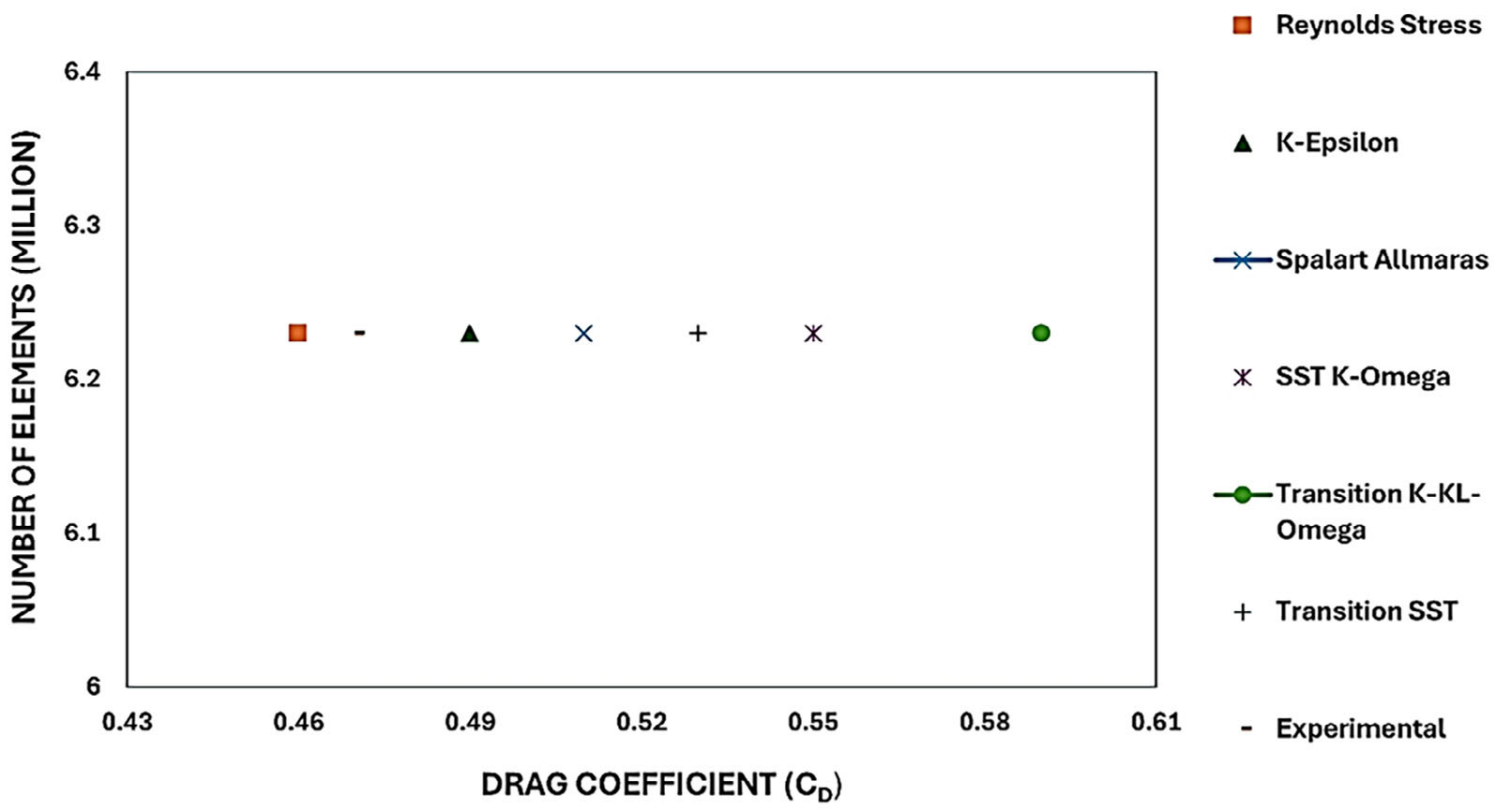
Turbulence model performance vs C _D_ for the best grid size.

Figure 12 shows the turbulence model test results for the baseline model. To study how the results varied for different models, six different models were used in this simulation. The obtained drag coefficient values for the different models in Table 5 were compared with the experimental drag coefficient in the previous study. ^18^ The Reynolds stress and K-Epsilon Standard models resulted in the lowest drag coefficient values.

**Table 5.** Turbulence model comparison results.

Viscous models	Obtained Drag coefficient (C _D_)	Experimental (C _D_) Reference value ^18^	Error (%)
Spalart Allmaras	0.51	0.47	8.51
K-Epsilon	0.49		4.25
SST K-Omega	0.55		17.02
Transition K-KL-Omega	0.59		25.53
Transition SST	0.53		12.76
Reynolds Stress	0.46		2.12

From Table 5, it can be observed that the Transition K-KL-Omega model resulted in a higher drag coefficient than the other models. The K-epsilon and Reynolds stress models had minimum drag values of C _D_ = 0.49 and C _D_ = 0.46, respectively, with the least error when compared with the experimental data. A comparison of the K-epsilon and Reynolds stress results will provide the best model for the final simulation.

Above Tables 6- 8, shows the flow contours and streamlines of the baseline truck for six different turbulence models compared with the reference results. Tables 9 and 10 compare the contour results of the best turbulence model.

**Table 6.** Comparison of velocity contour and streamline contour of reference paper result with different viscous model results. ^18^

Results	Reference paper results ^18^	K-Epsilon model	Reynolds stress model
**Velocity contour**	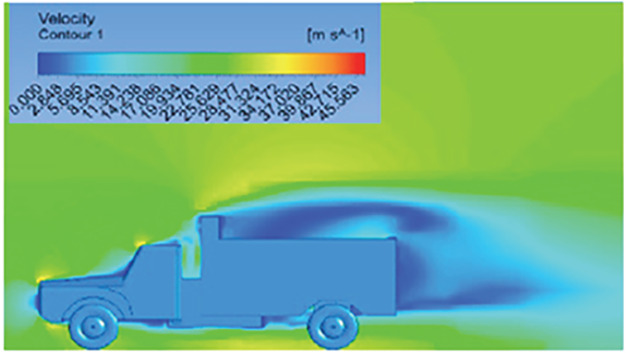 The contour shows a single flow separation point with a large low velocity high pressure wake region. The Red points indicate a high velocity region near the front hood and cabins leading edge.	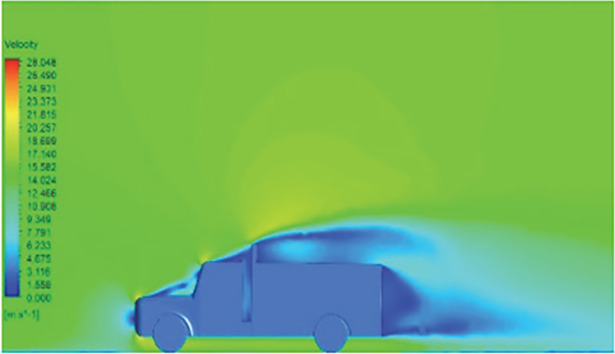 The velocity contour of K-Epsilon model shows a small low velocity region above the truck surface.	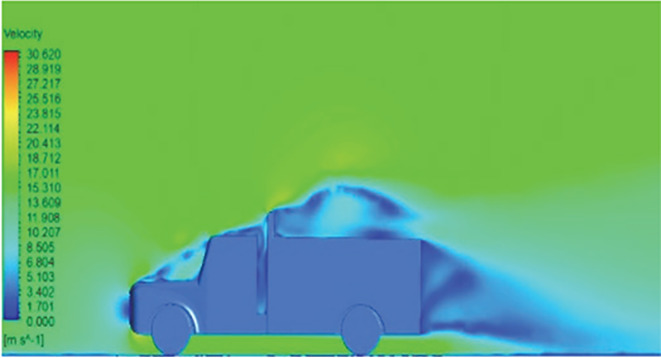 The velocity contour of this model shows a large disrupted low velocity region above the truck surface.
**Velocity streamline**	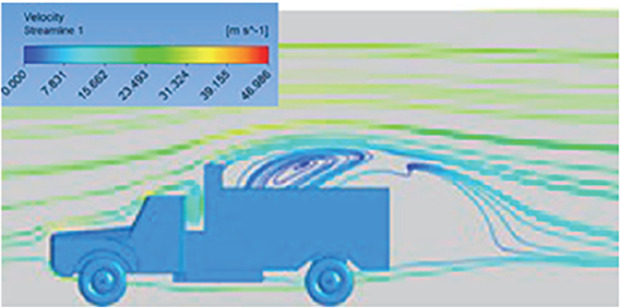 The reference paper streamlines show a large single circulating region above the truck. These regions contain small recirculating vortices which induce to drag.	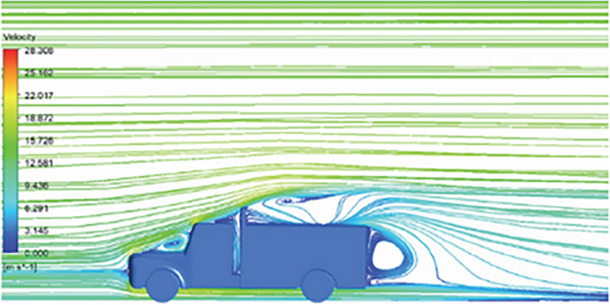 The streamlines obtained shows a small recirculating region between the cabin trailer Gap, small vortex formation occur at these gaps.	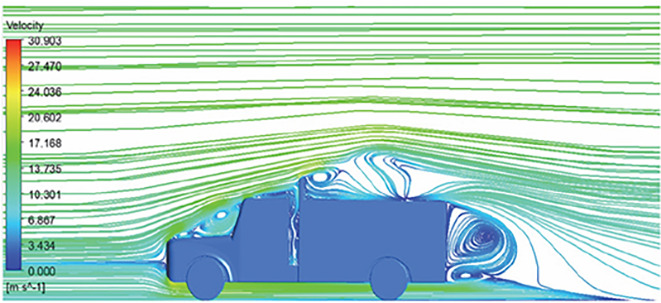 The streamlines are more detailed, three large recirculating vortices are formed. The Reynolds stress model has the best streamline compared to all other models.

**Table 7.** Comparison of velocity contour and streamline contour of reference paper result with different viscous model results. ^18^

Results	Reference paper results ^18^	Spalart Allmaras model	Transition SST model
**Velocity contour**	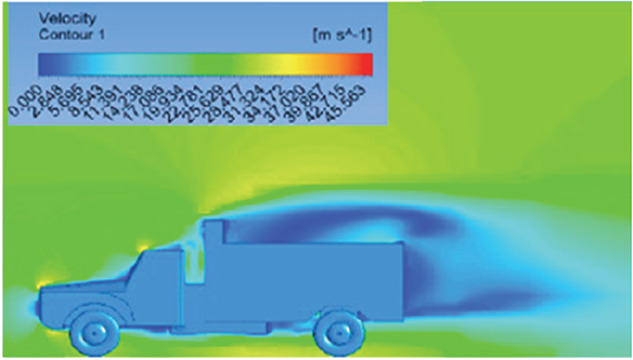 The contour shows a single flow separation point with a large low velocity high pressure wake region. The Red points indicate a high velocity region near the front hood and cabins leading edge.	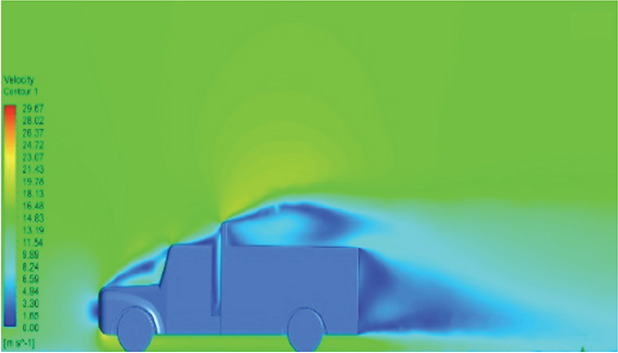 The velocity contour of Spalart Allmaras model shows a higher wake region above the truck surface.	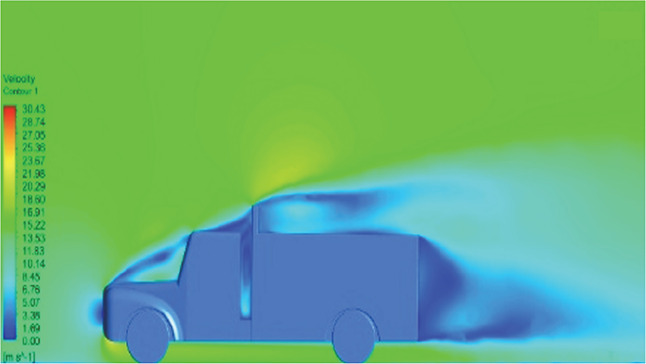 The above velocity contour shows an abrupt flow separation point near the leading edge of the trailer.
**Velocity streamline**	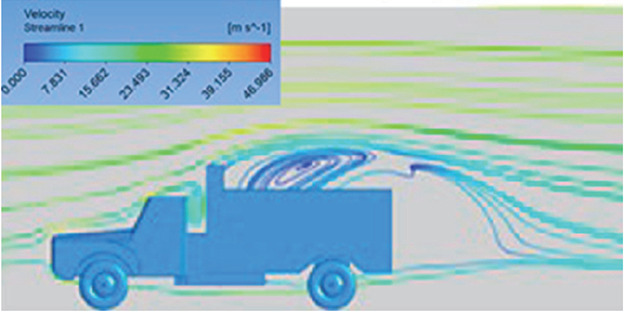 The reference paper streamlines show a large single circulating region above the truck. These regions contain small recirculating vortices which induce to drag.	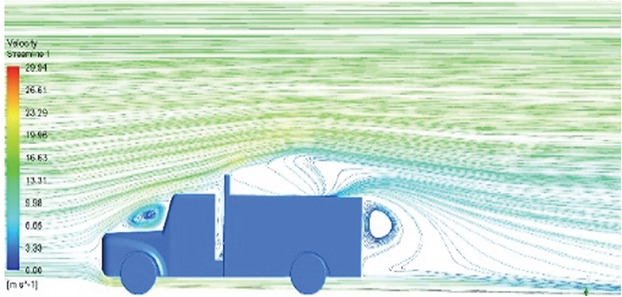 The streamlines show a large recirculating region at the front region near the windshield.	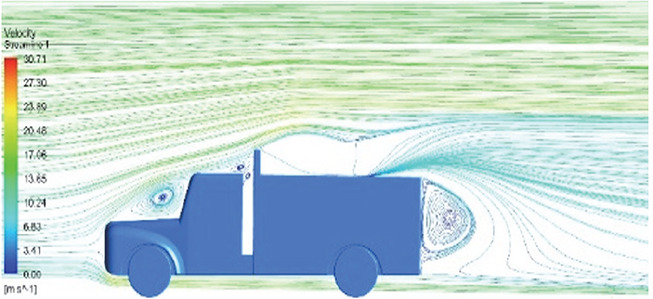 Two tiny vortices are formed in the gap region of the truck. The flow reattachment occurs far away.

**Table 8.** Comparison of velocity contour and streamline contour of reference paper result with different viscous model results. ^18^

Results	Reference paper results ^18^	SST K-Omega model	Transition K-KL-Omega
**Velocity contour**	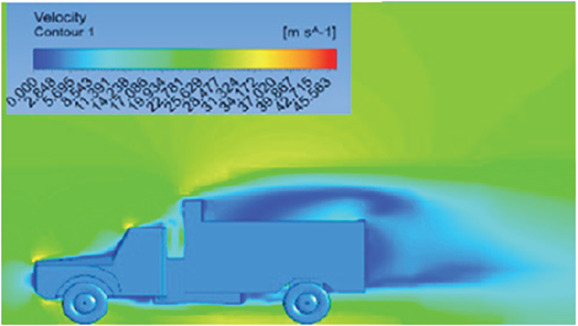 The contour shows a single flow separation point with a large low velocity high pressure wake region. The Red points indicate a high velocity region near the front hood and cabins leading edge.	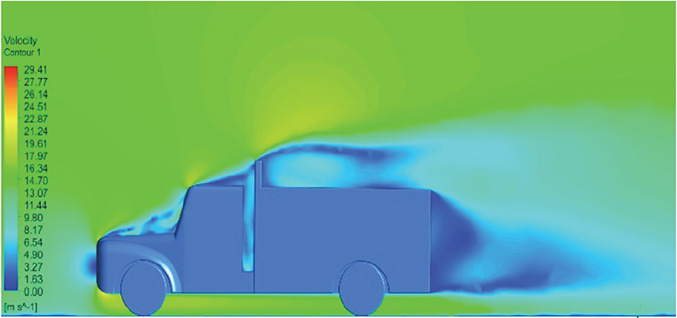 Abrupt air flow profile is observed near the frontal hood	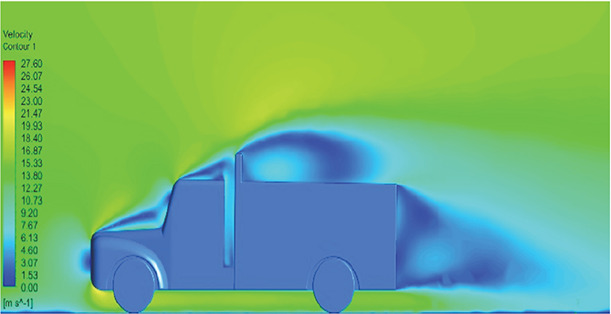 A high air velocity points are observed near the edges.
**Velocity streamline**	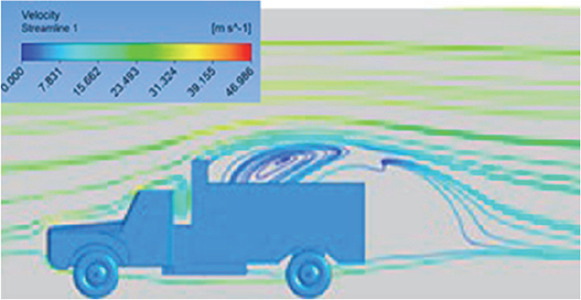 The reference paper streamlines show a large single circulating region above the truck. These regions contain small recirculating vortices which induce to drag.	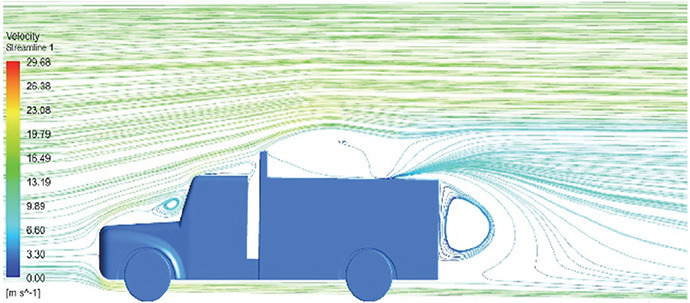 The streamlines obtained shows a small recirculating region at the front region near the windshield.	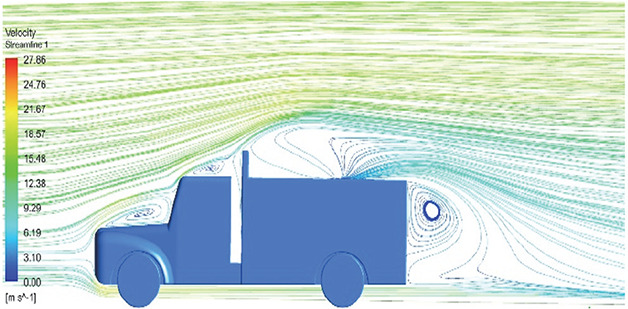 The streamlines obtained shows a large recirculating vortex at the rear end of the truck.

**Table 9.** Comparison of velocity contours.

Model	Velocity contour plot	Remarks
**K-Epsilon Standard**	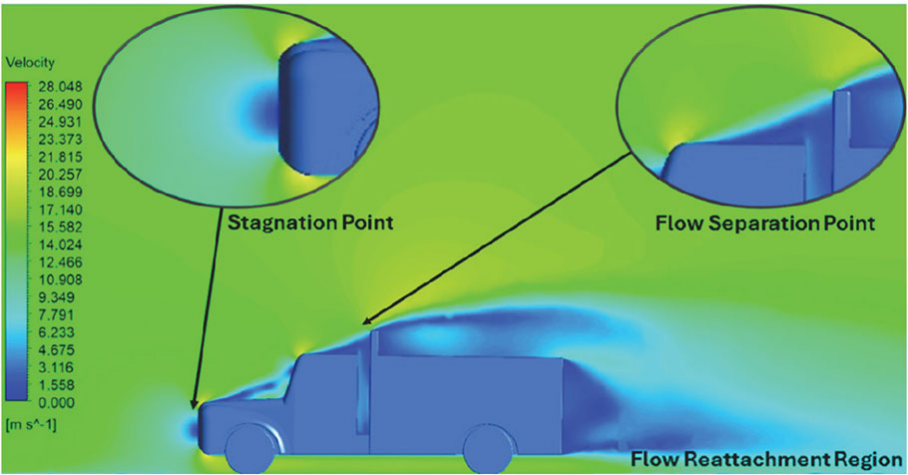 Figure 13: K-Epsilon model velocity contour.	A stagnation point at the front of the truck is observed. At this region the air velocity is zero and the pressure is at its maximum. A lower wake region is observed at the top of the truck. Flow separation points are clearly visible near the truck cabins leading edge.
**Reynolds Stress**	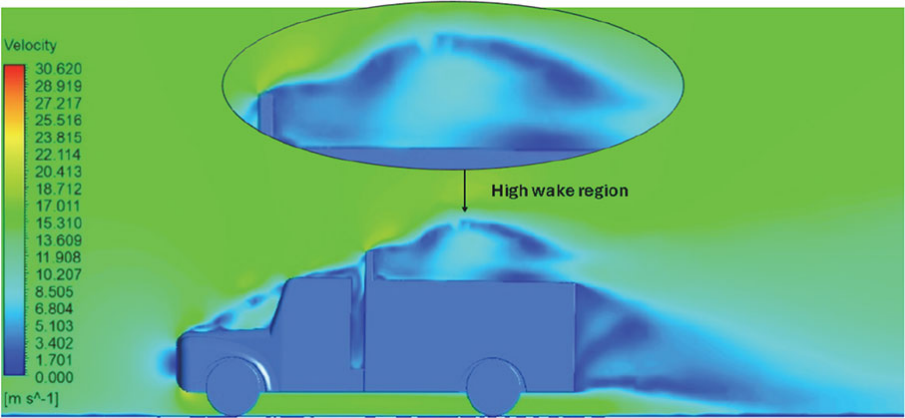 Figure 14: Reynolds Stress model velocity contour	Similar stagnation point is observed near front region of the truck. The low air velocity with a high-pressure wake region is much bigger in size due to bluff body, and an abrupt flow separation region is observed at the top of the truck.

**Table 10.** Comparison of velocity streamlines.

Model	Velocity streamline	Remarks
**K-Epsilon Standard**	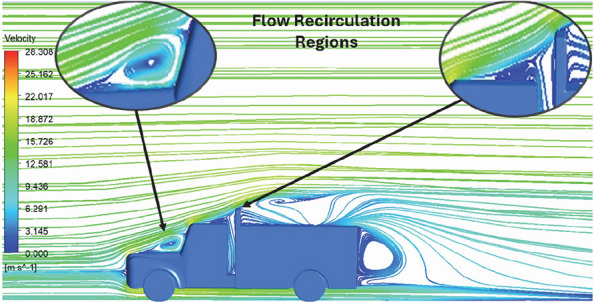 Figure 15: K-Epsilon model velocity streamline.	The detailed flow streamlines are obtained. The flow bubble near the gap between hood and windshield are visible. The recirculation in the gap region is prominent. Small vortex formations are observed near truck cabin.
**Reynolds Stress**	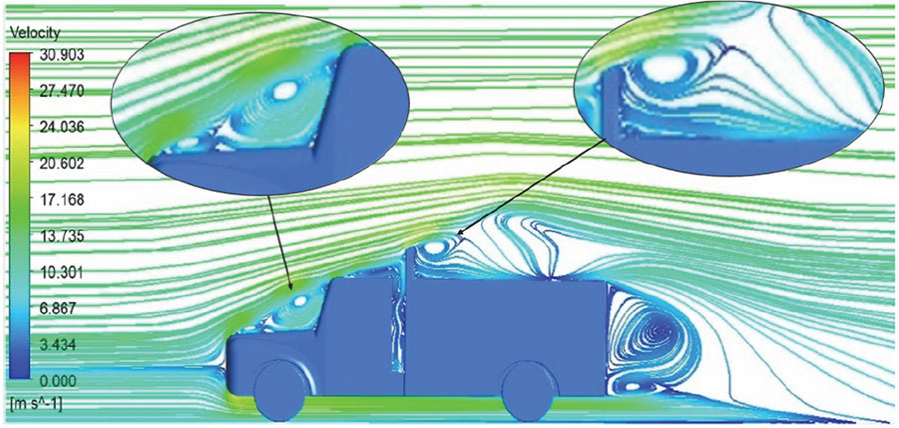 Figure 16: Reynolds Stress model velocity streamline.	This model yielded the best detailed streamlines. The small vortex formation near the hood is clearly visible. In the rear wake region both large and small vortex was captured. This streamline plot provides better flow visuals when compared to other models.

**Figure 13.**
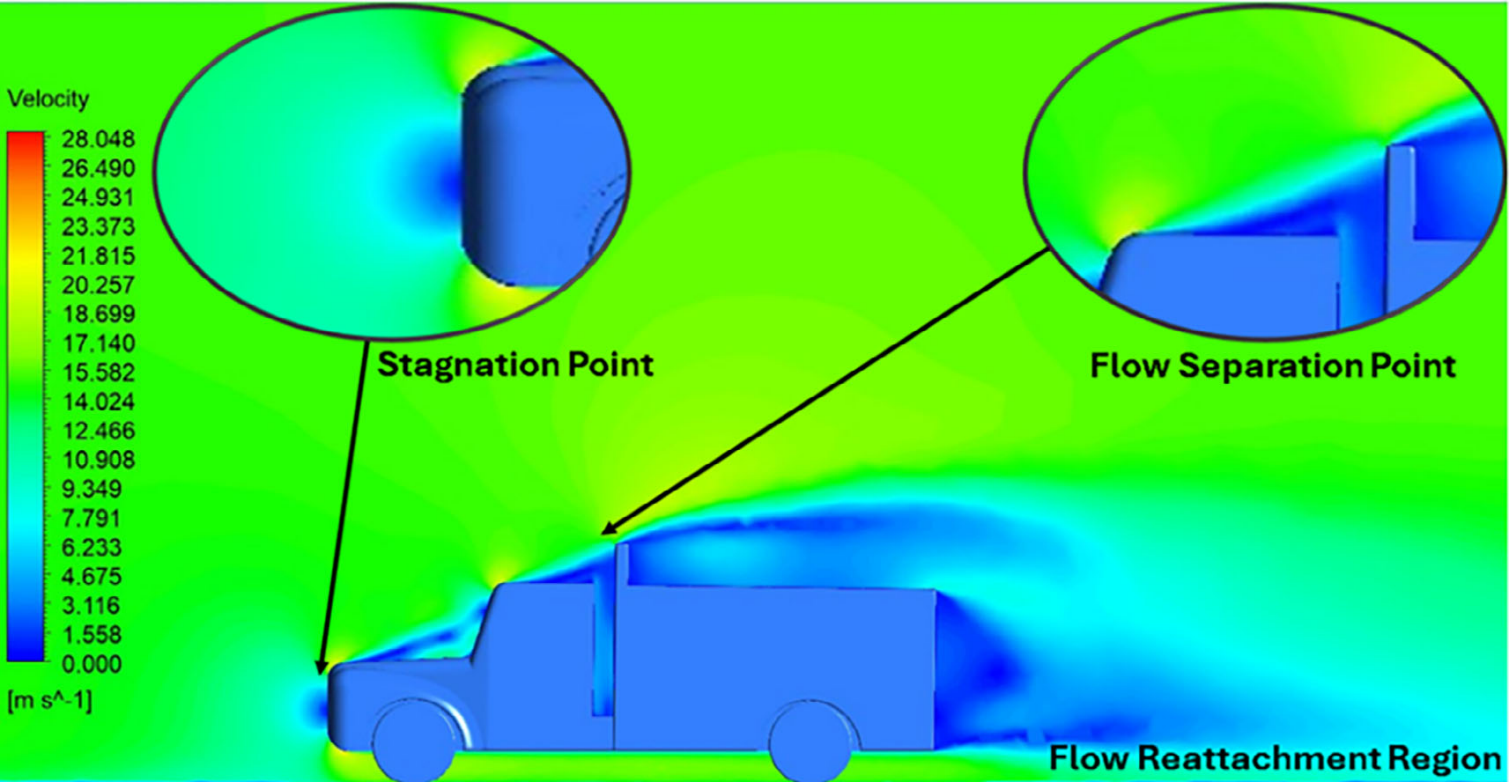
K-Epsilon model velocity contour.

**Figure 14.**
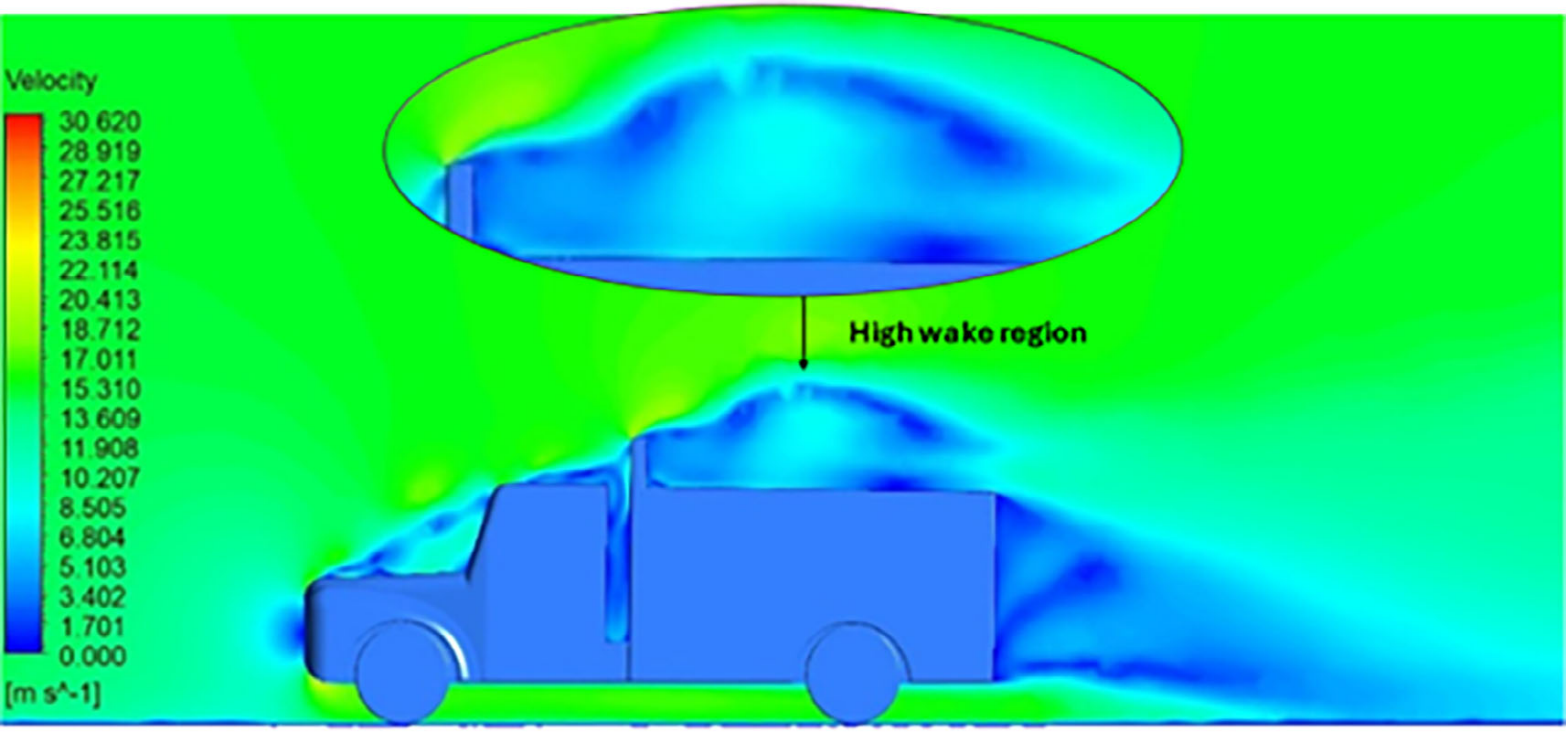
Reynolds stress model velocity contour.

The velocity streamlines in Figures 13 and 14 show the areas of flow recirculation of the K-epsilon and Reynolds stress models, respectively.

A significant area of flow recirculation is located at the back of the truck. Both large and small vortices were generated at these locations. The space between the cabin and the trailer produced a small flow recirculation area.

**Figure 15.**
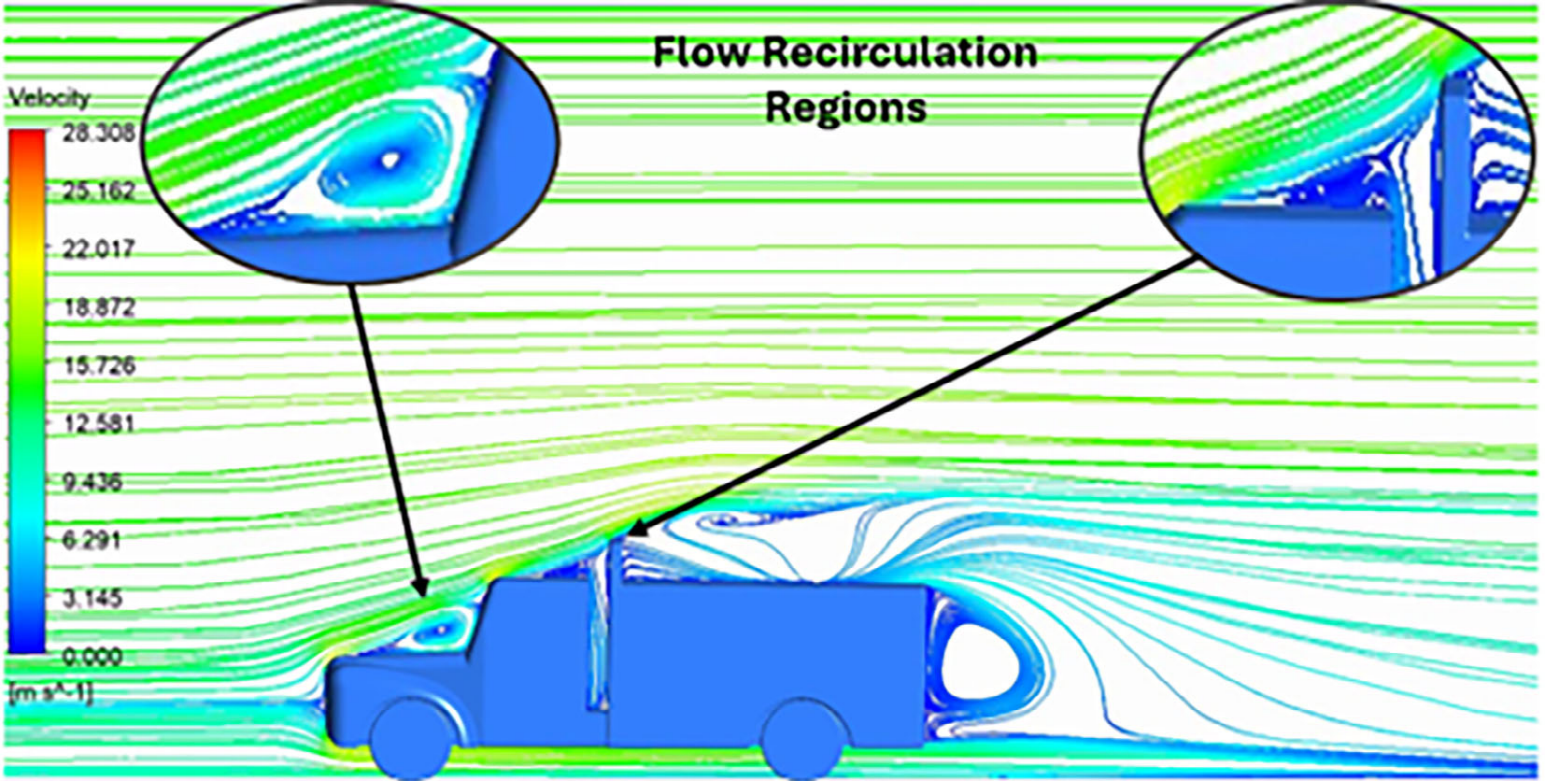
K-Epsilon model velocity streamline.

**Figure 16.**
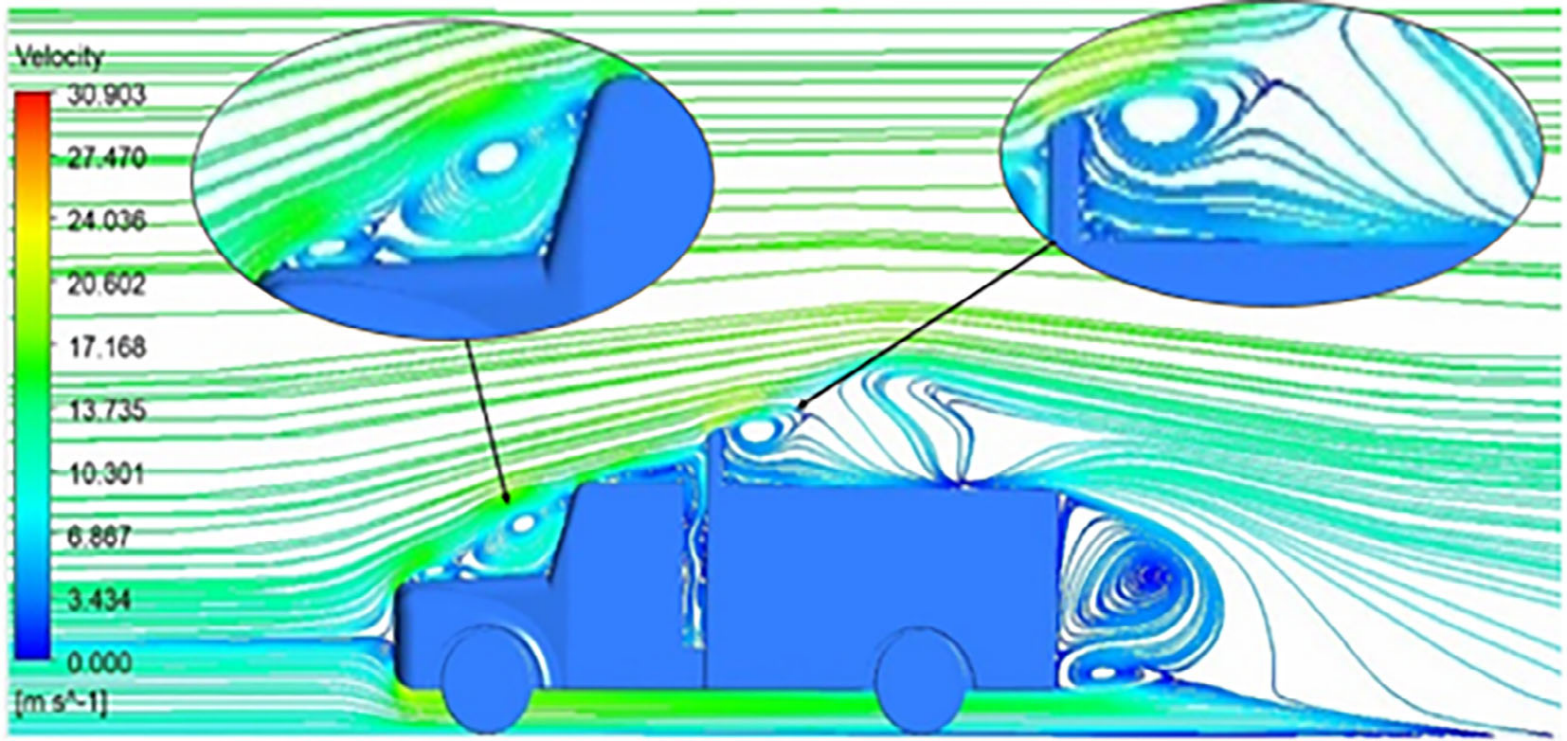
Reynolds stress model velocity streamline.

Introducing a gap enclosure allows air to flow from the sides of the truck cabin instead of flowing between the gaps. The underbody airflow is smooth in this case, and the vortex bubble formation near the front hood and cabin windshield is mainly caused by the angle between the hood and windshield ( Figures 15 and 16)

.

In conclusion, both of the above models resulted in accurate solutions and detailed streamlines. Considering the computational accuracy and cost, the K-epsilon (2 equation) model is much more efficient than the Reynolds Stress (7 equation) model.

The K-epsilon model is recognized for delivering precise outcomes under free-flow conditions, and this model results in faster solution convergence. Hence, The K-Epsilon Standard model was selected for the final simulation.

### 4.3 Varying inlet velocity

The inlet air velocities were varied to understand the variation in drag coefficient. As the vehicle speed increases, the drag also increases. The air velocity variation ranged from 40 to 90 km/h in the simulation with a 10 km/h increment. As the inlet air velocity increased, the drag coefficient also increased significantly.

From Table 11, it can be observed that for the velocity of 40 kmph (11.11 m/s) the drag coefficient value is 0.49 and for the maximum velocity, the drag coefficient is more than 1. Owing to the bluff body, the drag coefficient of a heavy truck vehicle was approximately 0.8. In this simulation, considering a highway operating speed of 70 km/h for a commercial truck, the drag coefficient obtained was C _D_ = 0.92.

**Table 11.** Values of drag coefficient for different air velocity.

Velocity (Kmph)	Drag coefficient (C _D_)
40	0.49
50	0.50
60	0.68
70	0.72
80	0.92
90	1.20

**Figure 17.**
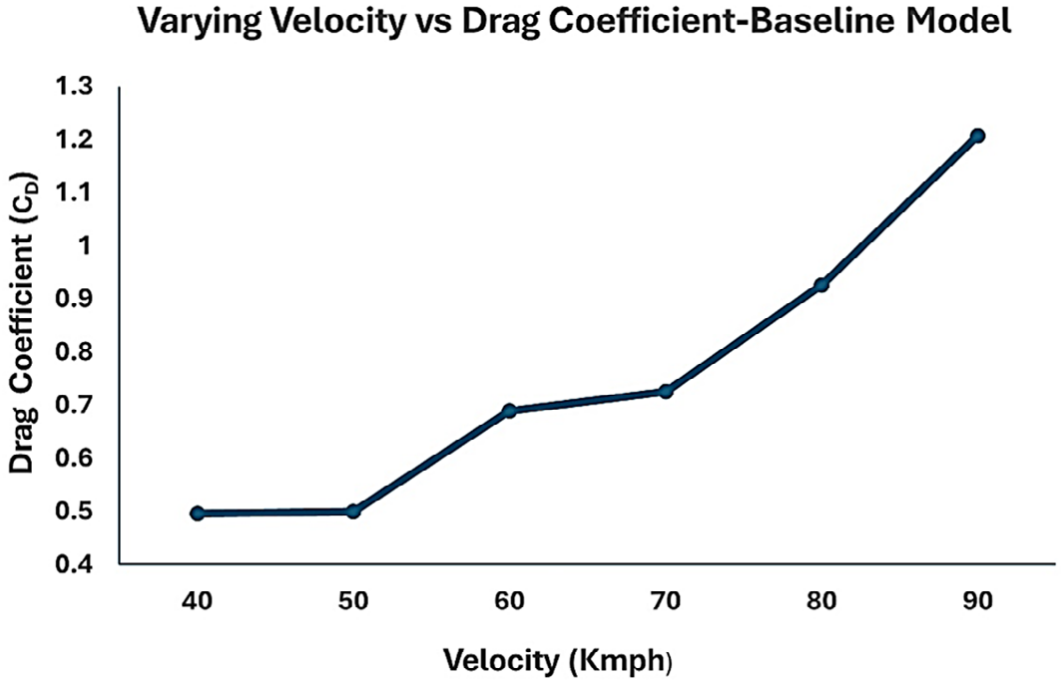
Varying velocity vs drag coefficient for baseline truck model.

From Figure 17, it can be observed that for an economical speed of 40-50 kmph, the drag values are minimal. As the speed increased, the drag suddenly increased. The highway operating speed of a truck is 70-80 kmph. At this speed, the fuel consumption is very high, and minimizing the overall drag experienced by the truck will help reduce fuel consumption. The overall drag coefficient was significantly higher in this case. At the maximum operating speed of the truck, the drag value exceeded 1, which is uncommon. Hence, by streamlining the body of the truck, a lower drag coefficient can be obtained. Generally, the drag value for these trucks is approximately 0.8.

**Figure 18.**
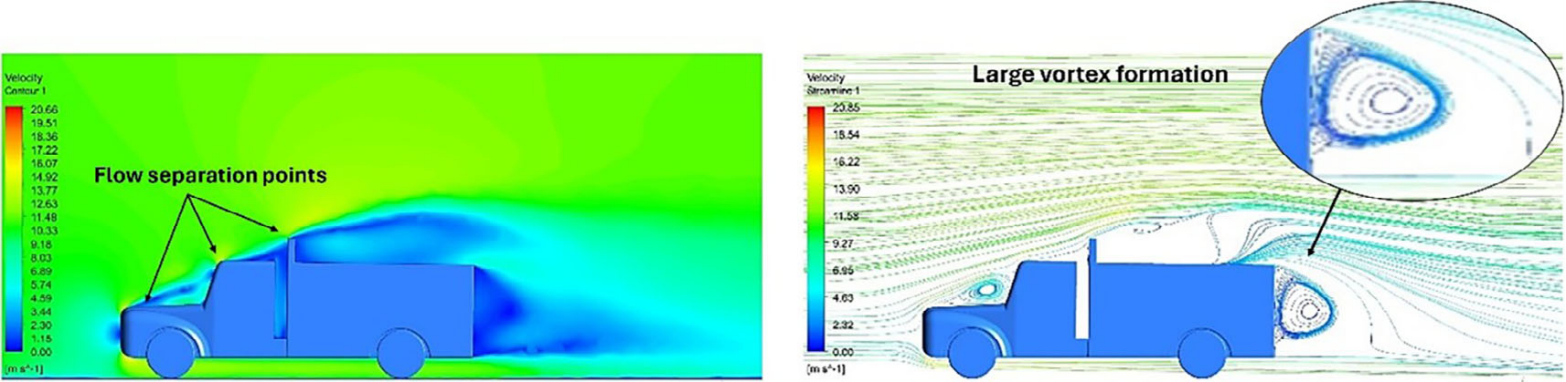
Velocity contour and velocity streamline at 40 Kmph.

**Figure 19.**
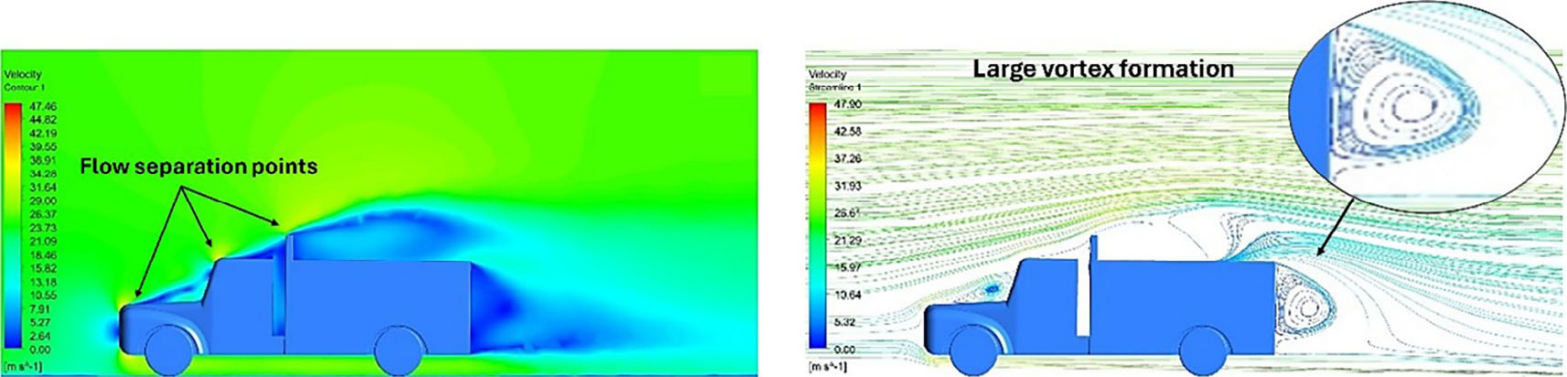
Velocity contour and velocity streamline at 90 Kmph.

Figures 18 and 19 show the velocity contours and streamlines for the maximum and minimum air velocities, respectively, from this simulation. Similar flow contours were obtained for two large wake regions near the rear end of the truck.

### 4.4 Baseline model comparison and validation

The velocity contours and streamlines were compared with respect to the reference paper. A similar flow contour was obtained for the baseline truck analysis, and the results were satisfactory. The obtained drag coefficient value was closer to the experimental data, with the least error. Results were obtained and studied by varying the inlet boundary conditions. Finally, the K-epsilon model was selected as the best turbulence model.

**Figure 20.**
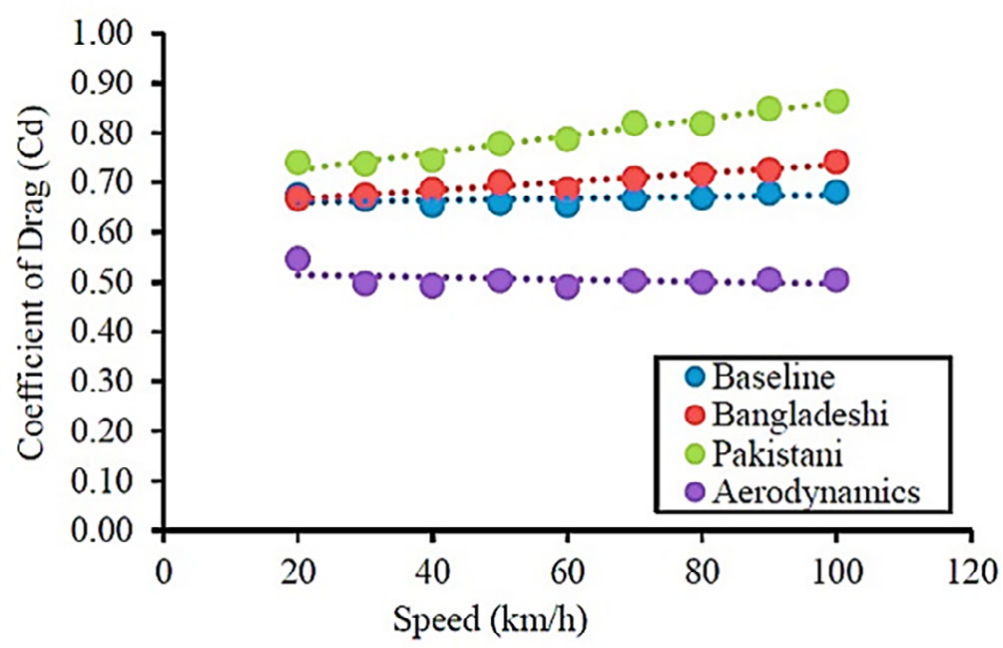
Drag coefficient (C _D_) as a function of wind speed. ^18^

**Figure 21.**
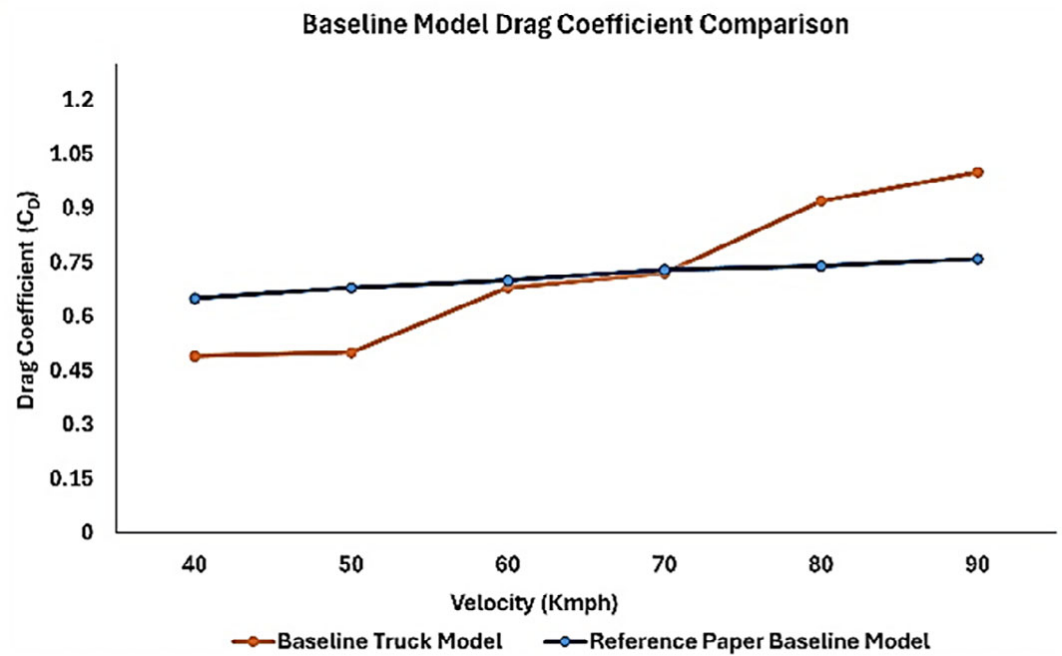
Drag coefficient (C _D_) vs varying velocity, comparison of reference paper ^18^ model and present work baseline truck model results.

In Figure 20, The baseline model data are plotted in blue in Figure 20. The overall drag coefficient for varying speeds was approximately C _D_ = 0.7. The comparison graph in Figure 21 shows the variation in drag values for different air speeds between the two models.

From Figure 21, it can be observed that for wind speeds of 70 km/h and above, the drag coefficient value increases significantly for the baseline model (orange line) in the present work. The reference paper baseline model drag (C _D_) value is between 0.6 and 0.75. The overall drag coefficient value was less than that of the present work. In Figure 20, for the aerodynamically optimized truck model, the graph shows an overall drag coefficient value between 0.45 and 0.55. In the experimental reference paper, the installation of aerodynamic fairing is very effective, as the result shows a 12% reduction in drag forces compared to the baseline model. ^18^ Because detailed values for the drag coefficient are not provided in the experimental reference paper, by referring to Figure 20, the drag values between 0.6 the purposes considered for comparison and validation purposes. The aim of this study is to improve aerodynamic efficiency.

The minimum error obtained by comparing the reference paper result and the present simulation paper result for an average speed of 60 km/h was 2.86%. The baseline model results were accurate, and the obtained drag value was closer to the experimental reference value. ^18^

### 4.5 Optimised model analysis

**Figure 22.**
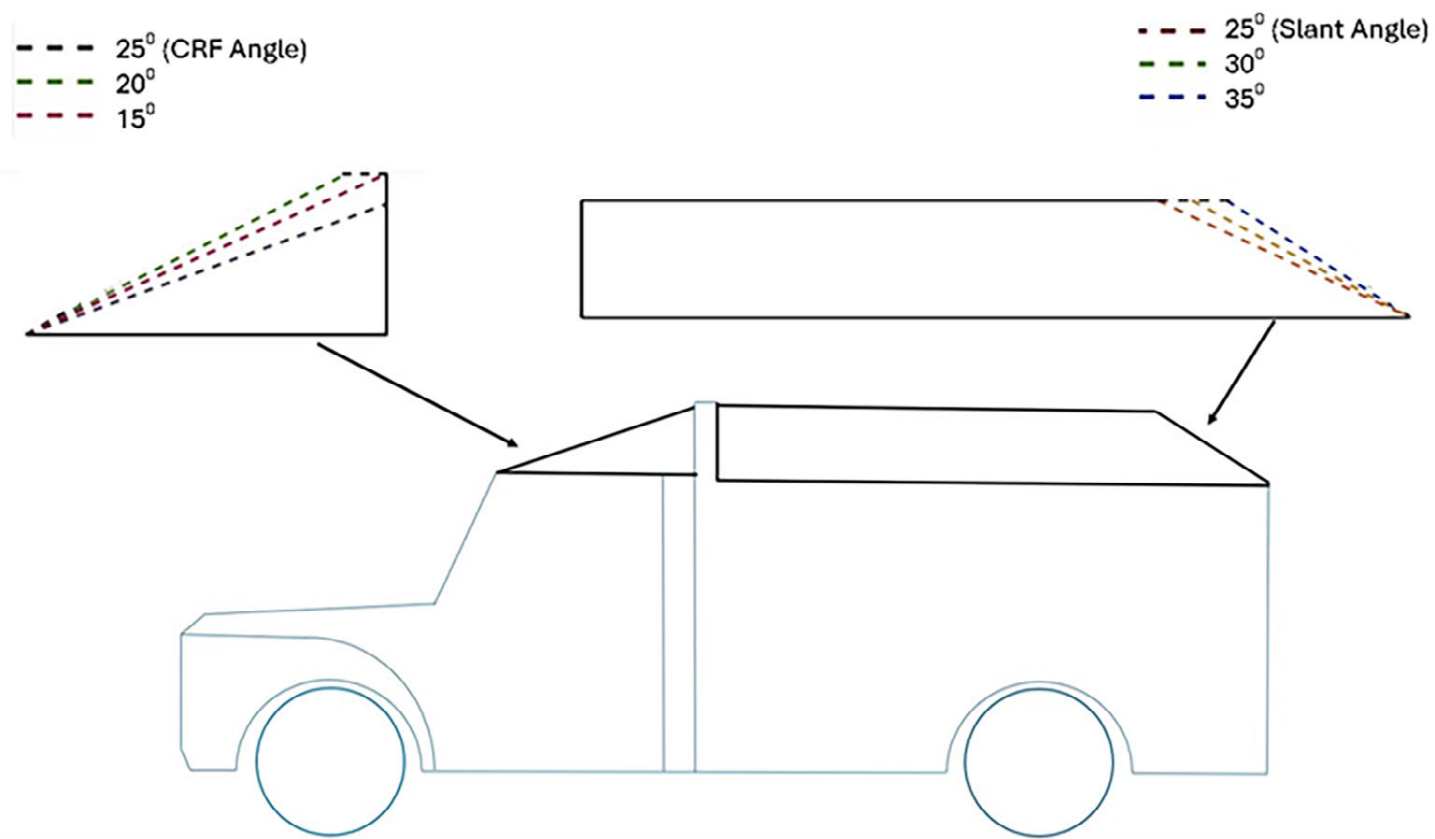
Various CRF and square back design modifications for the baseline truck.

The Baseline truck model was redesigned, as shown in Figure 22, with a total of three design modifications: CRF was fitted to the cabin roof, a gap enclosure was added, which was then combined with CRF, and the square back was modified with an optimal slant angle. Various CRF angles were analyzed, and the best CRF angle was chosen for the final simulation. Similarly, various square-back slant angles were analyzed, and the best slant angle model was chosen. Finally, the best aerodynamically optimized truck design was presented.

For the rear square-back modifications of the truck, three different slant angles with 5° variation for the square-back were used for the analysis.

For each modification, a grid-independence test was conducted to ensure that an optimal mesh was obtained. For the best grid size, various turbulence models were tested, and for the best model with the least computational error, a final simulation with varying air velocity was conducted. The results of all optimizations were then compared to obtain the best model. Finally, an aerodynamically optimized truck model is presented.

**Figure 23.**
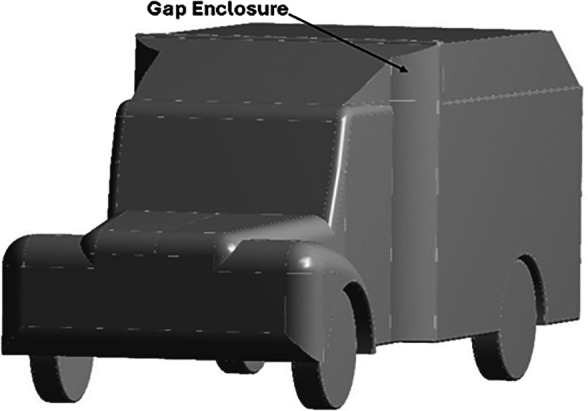
Optimised truck model (Isometric view).

**Figure 24.**
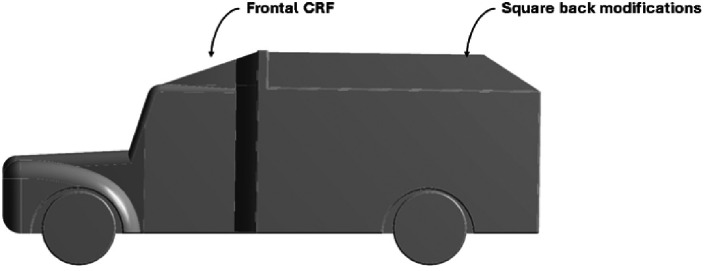
Optimised truck model (Side view).

Figures 23 and 24 show the 3D models of the optimized truck. The truck’s geometrical dimensions are the same as those of the baseline, as given in Table 1. The dimensions of the CRF, Gap enclosure, and squareback for all modifications are listed in Tables 12, 13, and 14, respectively.

**Table 12.** CRF model dimensions.

**Model 1** **α = 25°** W1 = 0.1457 m W2 = 0.1590 m X = 0.088 m X1 = 0.025 m Y = 0.04 m	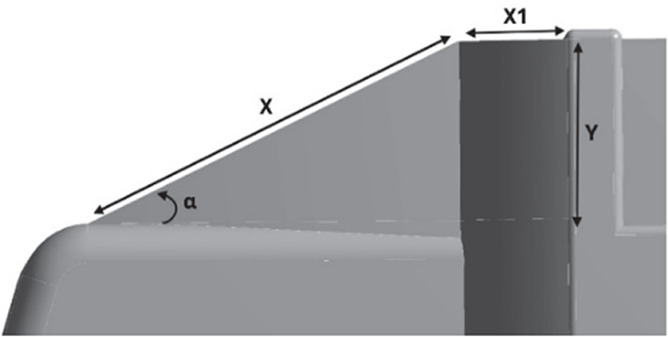	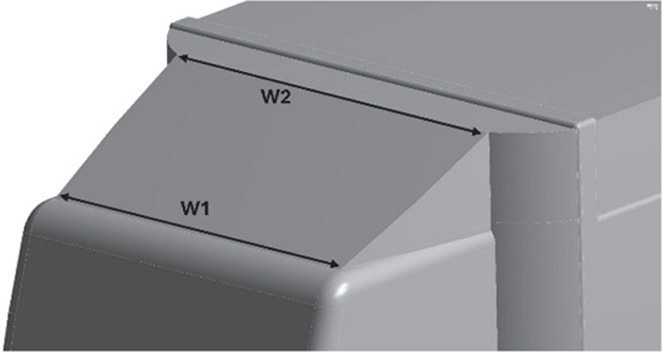
**Model 2** **α = 20°** W1 = 0.1457 m W2 = 0.1590 m X = 0.11063 m Y = 0.04 m	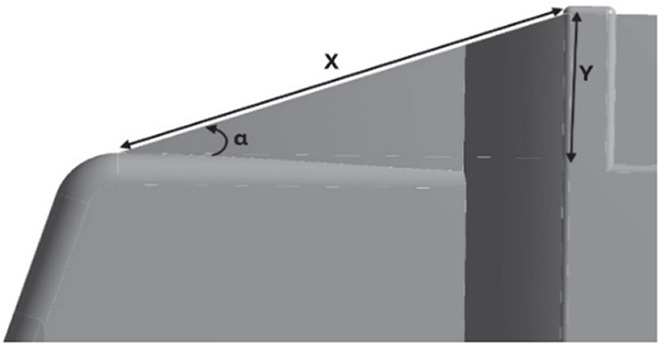	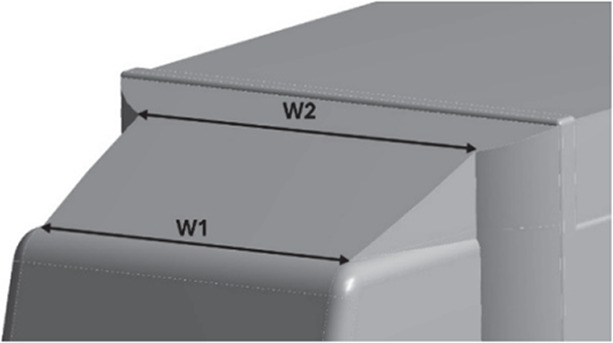
**Model 3** **α = 15°** W1 = 0.1457 m W2 = 1590 m X = 0.11063 m Y = 0.04 m	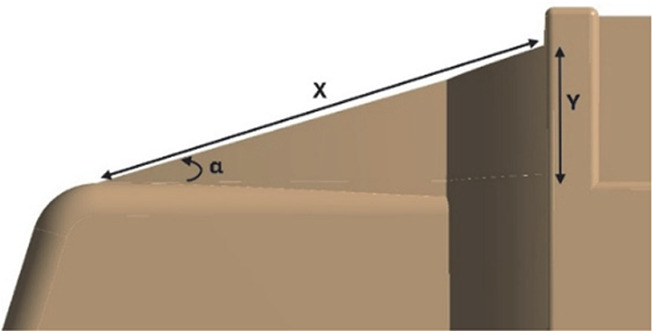	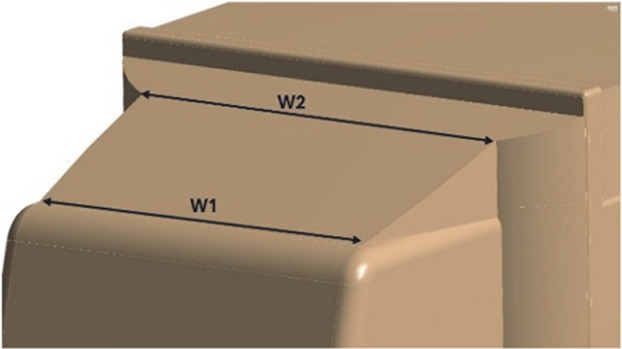

**Table 13.** Gap fairing dimensions (1/10 ^th^ scale).

Gap Enclosure Length
L1 = 0.025 m
L2 = 0.025 m
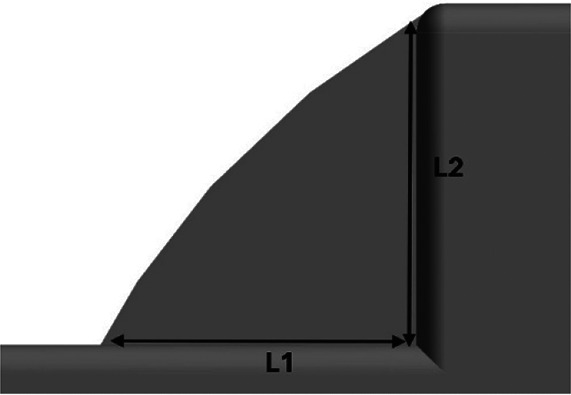

**Table 14.** Square back modification dimensions (1/10 ^th^ scale).

25° Slant angle	β = 25°, Y = 0.04 m, L1 = 0.3 m, L2 = 0.2107 m, L3 = 0.0900 m
30° Slant angle	β = 30°, Y = 0.04 m, L1 = 0.3 m, L2 = 0.2323 m, L3 = 0.0718 m
35° Slant angle	β = 35°, Y = 0.04 m, L1 = 0.3 m, L2 = 0.2382 m, L3 = 0.0670 m

**4.5.1 Cab roof fairing geometry**

Aerodynamic devices, such as frontal wind deflectors and gap fairings, are fixed devices that do not require external power to operate and are known as passive drag reduction devices. These are the most effective devices for bluff bodies, such as trucks. These wind deflectors, commonly known as ‘Cab Roof Fairing’ (CRF), help the vehicle move smoothly through the air by redirecting the airflow around the vehicle. At higher speeds, these devices improve vehicle stability and reduce the side force under crosswind conditions. ^7^ Similar studies were conducted using different CRF designs.

**Figure 25.**
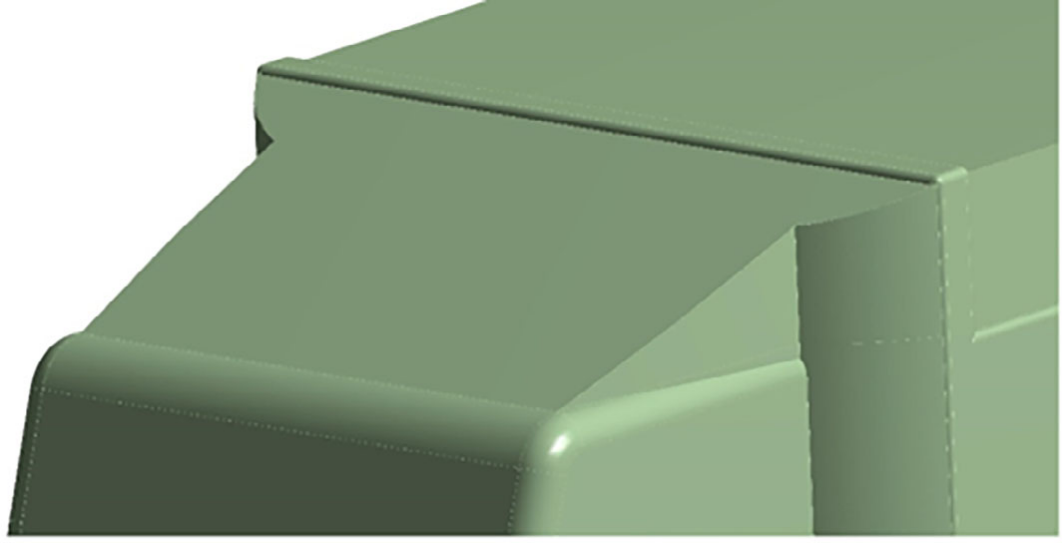
CRF attached with truck cabin and gap enclosure.

Figure 25 shows the optimized CRF attached to the truck cabin. Three different CRF models with different frontal angles were modelled. The CRF geometry was modelled using the ANSYS DesignModeler software and was attached to the baseline truck model cabin.

**4.5.2 Gap fairing geometry**

Additionally, to reduce flow recirculation in the gap between the truck cabin and goods trailer, a gap fairing was designed and combined with the CRF. The combination of CRF and gap farming ensures an optimal frontal design and enhances the appearance of trucks. For a truck cabin-trailer gap of 0.02 m, the gap enclosure was designed to avoid recirculating airflows. The dimensions are presented in Table 13.

**4.5.3 Square back geometry**

**Figure 26.**
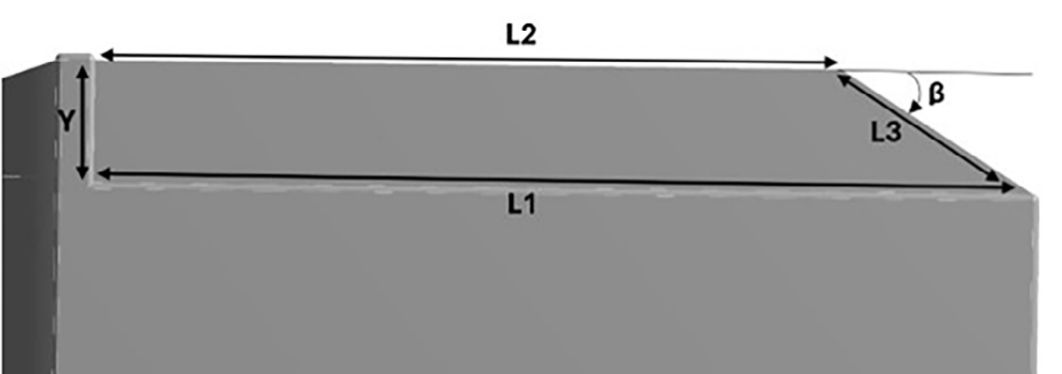
Square back geometry (Side view).

Figure 26 shows the squareback modifications. The squareback modifications were inspired by the Ahmed Body squareback model. The Ahmed Body model is the most widely used model in the research field. In this study, ^21^ a study on simplified three-dimensional square back Ahmed model was developed. This study signifies the importance of the slant angle (β), where β = 30° is known as the critical angle. Many researchers have conducted experimental and numerical analyses on the Ahmed model. The aim was to maintain airflow close to the body to achieve a minimal wake zone.

**Figure 27.**
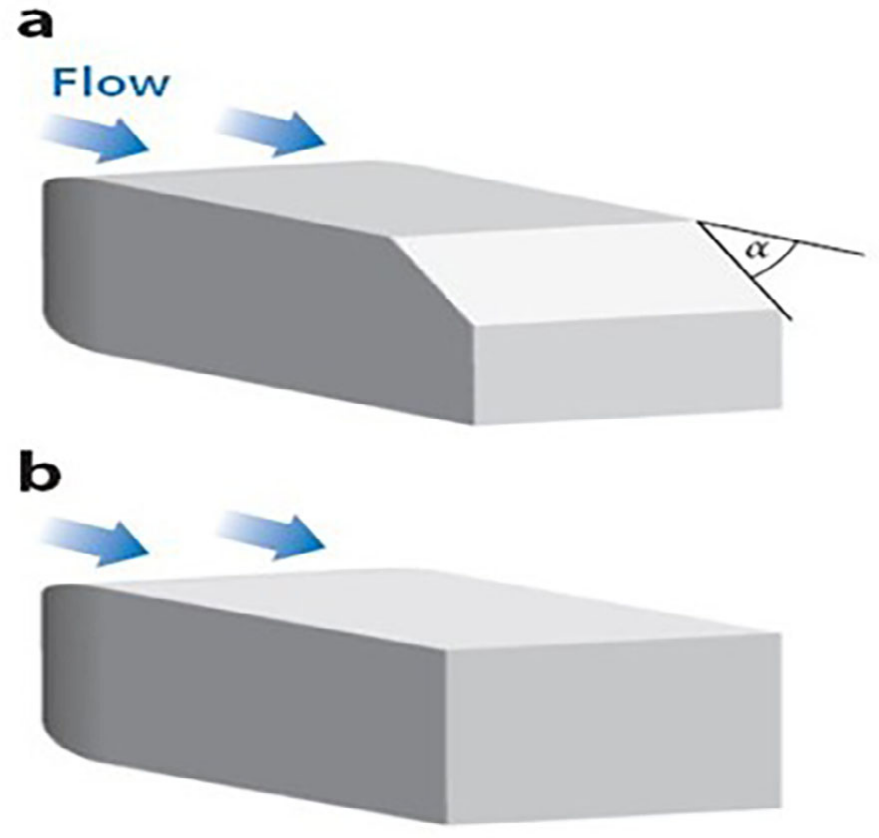
Simplified three-dimensional square back Ahmed model. ^21^

Figure 27 shows a simplified three-dimensional squareback Ahmed model. The current model demonstrated significant success in flow modification, with the angle adjusted between 25° and 35°. A Grid independence test was performed for all simulations, along with the evaluation of different turbulence models. The optimal model was then used to analyze the results due to changes in air velocity. Ultimately, the slant angle that minimized the overall drag was selected as the final model. The flow contours associated with each modification were thoroughly examined and analyzed.

**4.5.4 Optimised model results**

**Figure 28.**
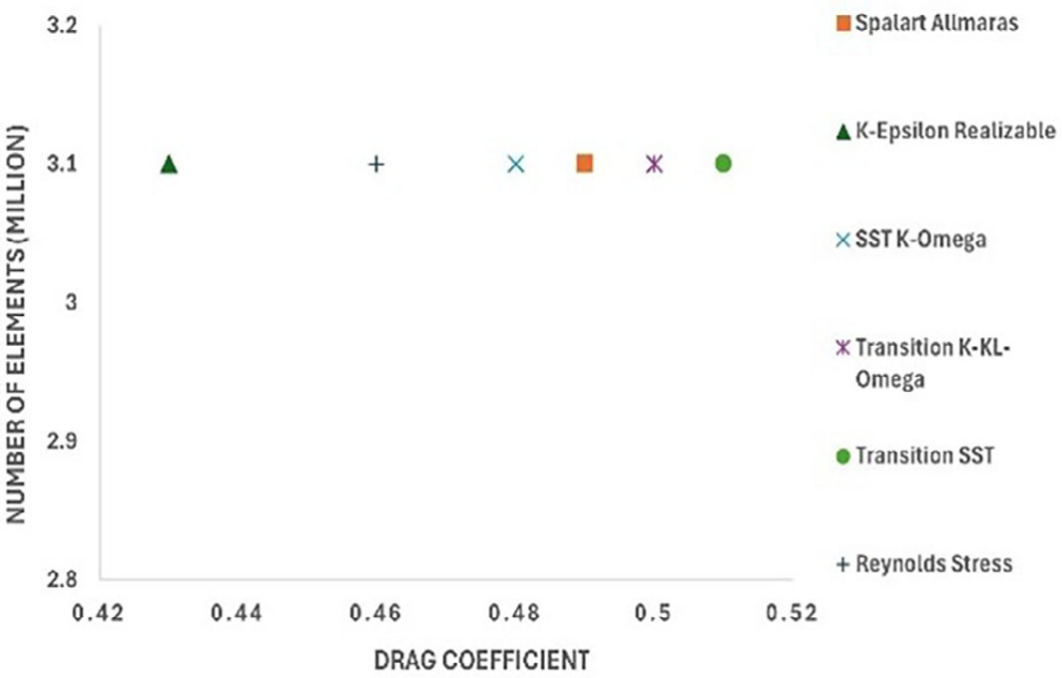
CRF model 1: Turbulence test.

**Figure 29.**
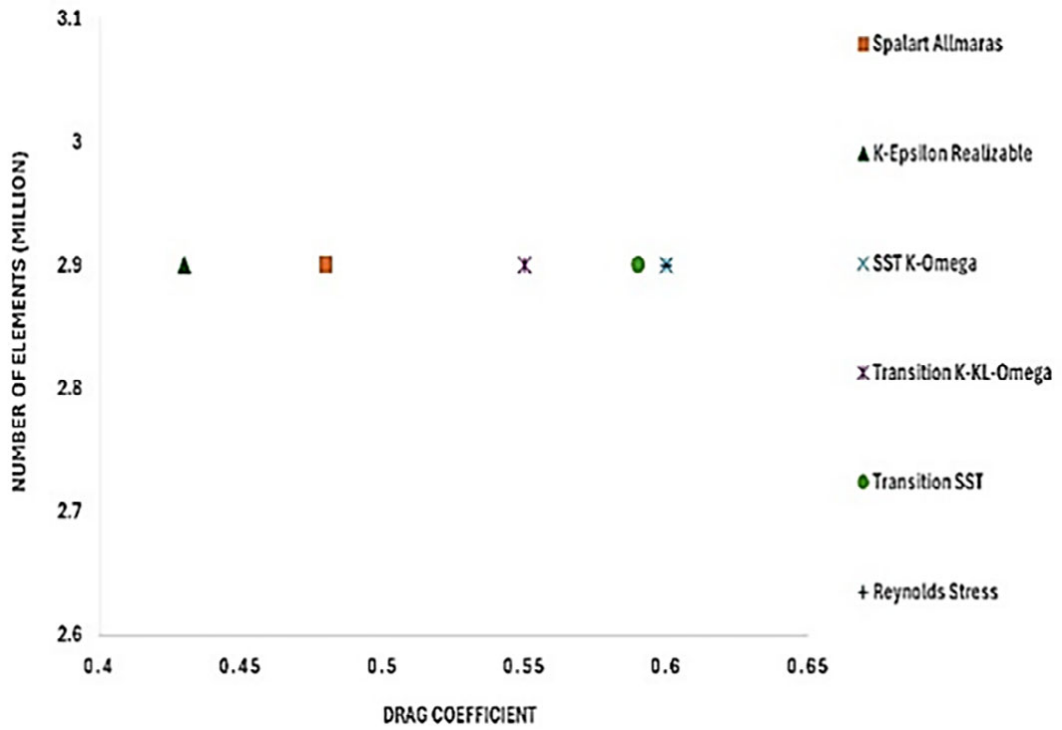
CRF model 2: Turbulence test.

After performing the grid analysis for each modification, the best grid was chosen for turbulence model testing. From Table 15 ( Figures 28 and 29), it can be observed that the K-Epsilon Realizable model resulted in the lowest drag values in Models 1 and 2. Using K-Epsilon Realizable model, the air velocity was varied in the simulation, and the obtained results were analyzed.

**Table 15.** CRF turbulence model test results.

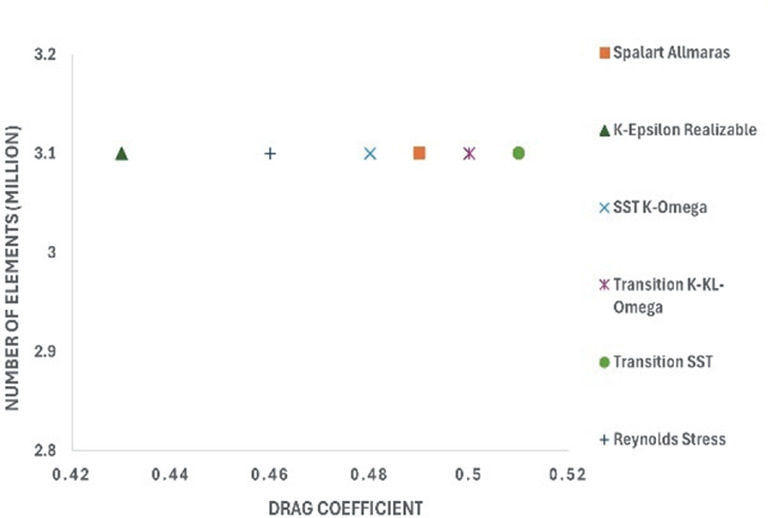 Figure 28: CRF model 1: Turbulence test	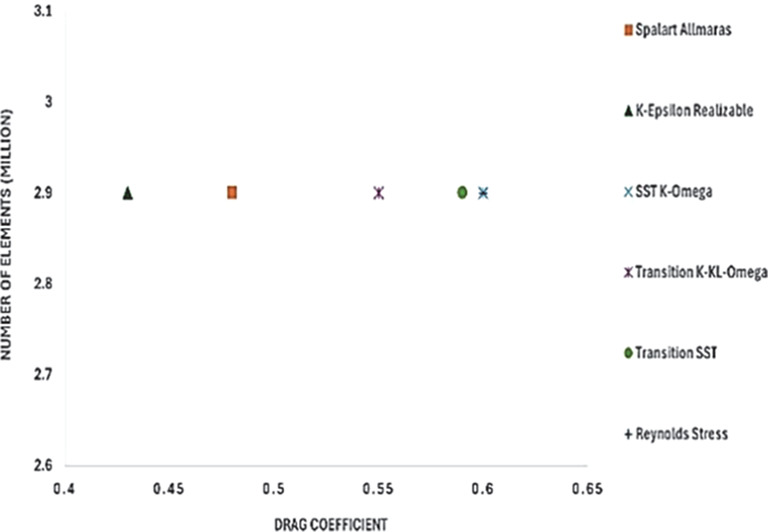 Figure 29: CRF model 2: Turbulence test
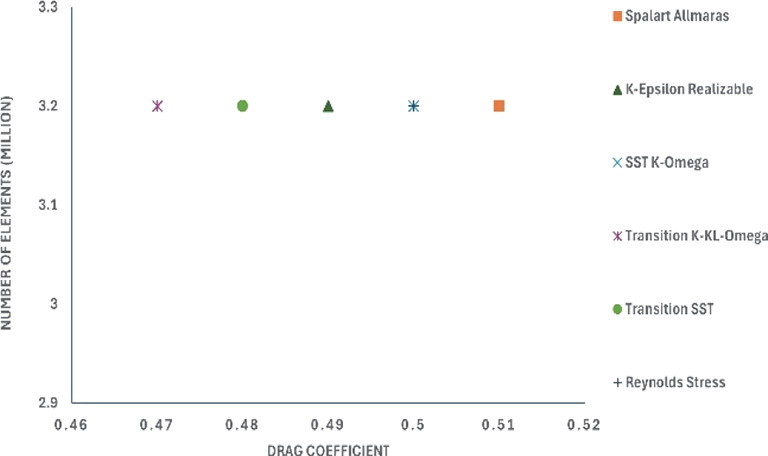 Figure 30: CRF model 3: Turbulence test	

The 30° slant angle model resulted in a higher C _D_ value compared to the other models, but for varying air speeds, the drag value was almost constant. An optimal angle for the square back maintains the airflow attached to the surface.

A smaller wake region was observed in this case (refer to Table 16). The overall drag value does not vary as the air speed increases. The 30° slant angle model proved to be an effective device for reducing the drag under high-speed conditions.

**Table 16.** Comparison of velocity contours of baseline and optimized models.

Baseline model	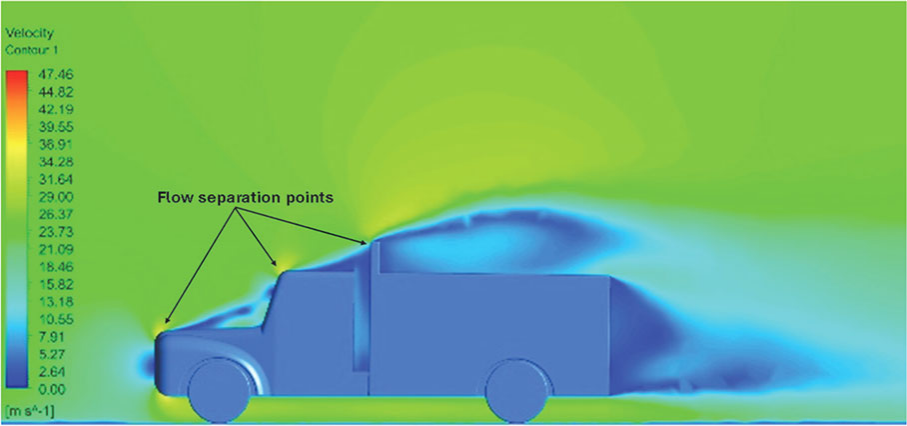
Optimised model	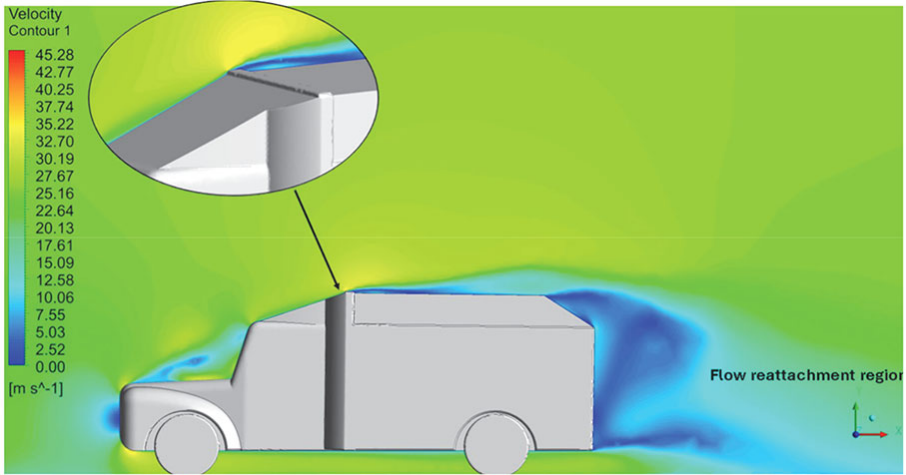

CRF model 2 exhibited the lowest overall drag when compared to the other models. The effective angle of the front wind deflector reduces drag accumulation in the frontal region of the truck. CRF Model 2 is the best wind deflector model for trucks. The optimal angle and height of the deflector resulted in smooth airflow above the truck.

**4.5.5 Comparative analysis**

Table 16 compares the velocity contours of the baseline and optimized models. A small wake region was observed in the optimized model. Flow attachment occurs quickly in the optimized model. The low air velocity regions (blue shaded regions) are more streamlined in the optimized model. Abrupt flow separation was observed in the baseline model.

Table 17 compares the velocity streamlines of the baseline and optimized models. The air flow recirculation regions were reduced in the optimized model. The gap enclosure directs the airflow smoothly around the truck. A large vortex formation was observed in both cases.

**Table 17.** Comparison of velocity streamlines of baseline and optimized models.

Baseline model	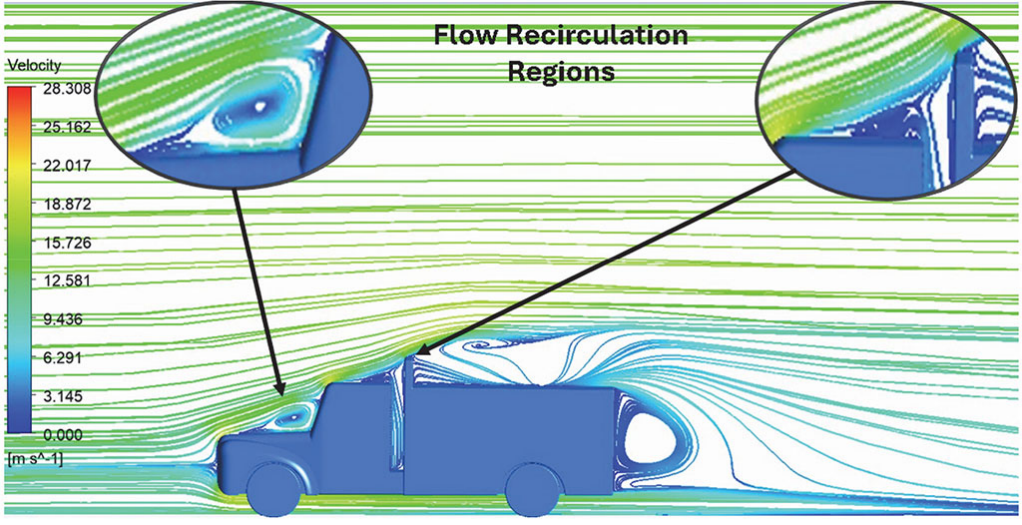
Optimised model	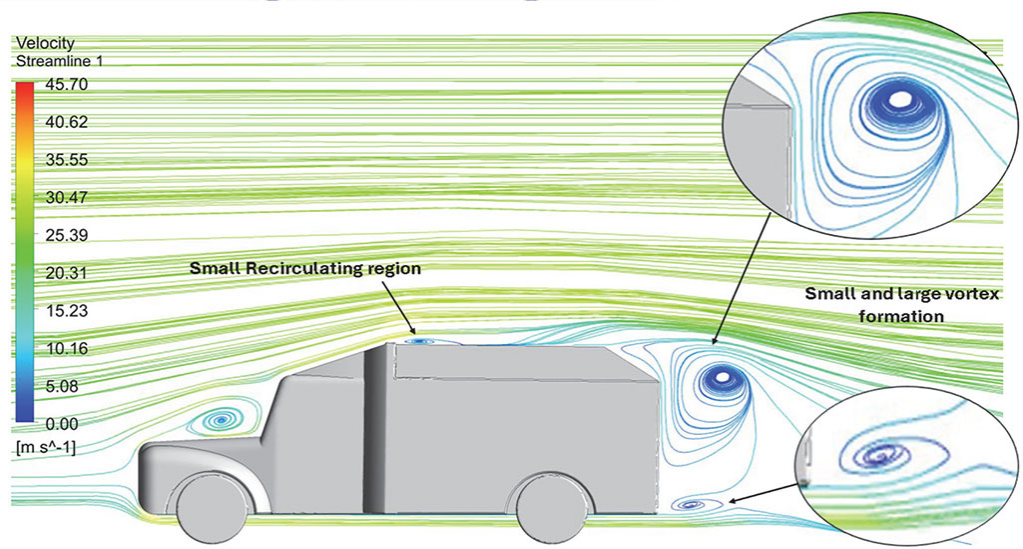

small-scale vortex formations are minimized in the optimized model, thereby improving the air flow around the truck model.

Table 18 compares the surface pressures of the baseline and optimized models. The Trailer body surface pressure was minimized in the optimized model. The pressure ranges from -525 Pa to 146 Pa. The frontal area experienced the highest pressure per unit area. The CRF and gap enclosures were effective in minimizing the surface pressure near the trailer body.

**Table 18.** Table of comparison for surface pressure of baseline and optimized models.

Baseline model	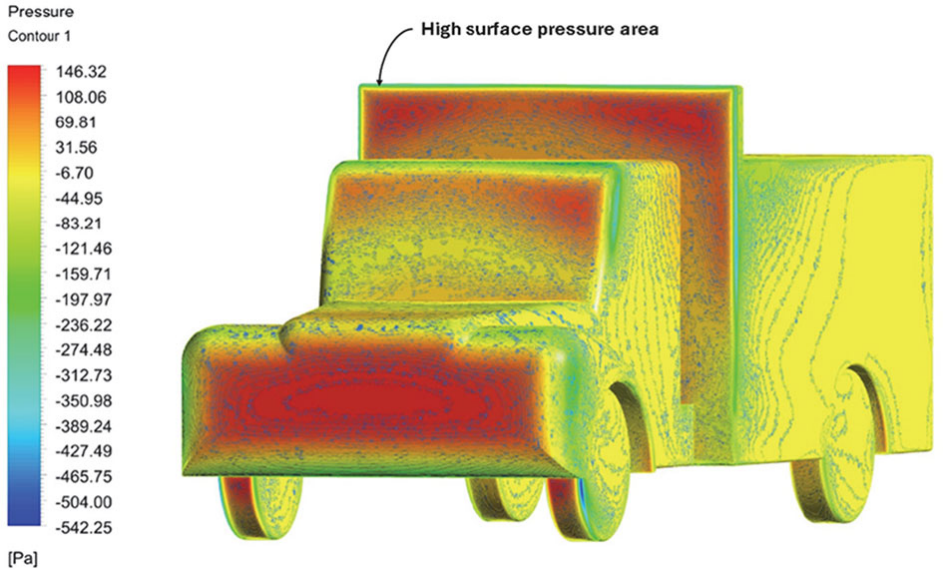
Optimised model	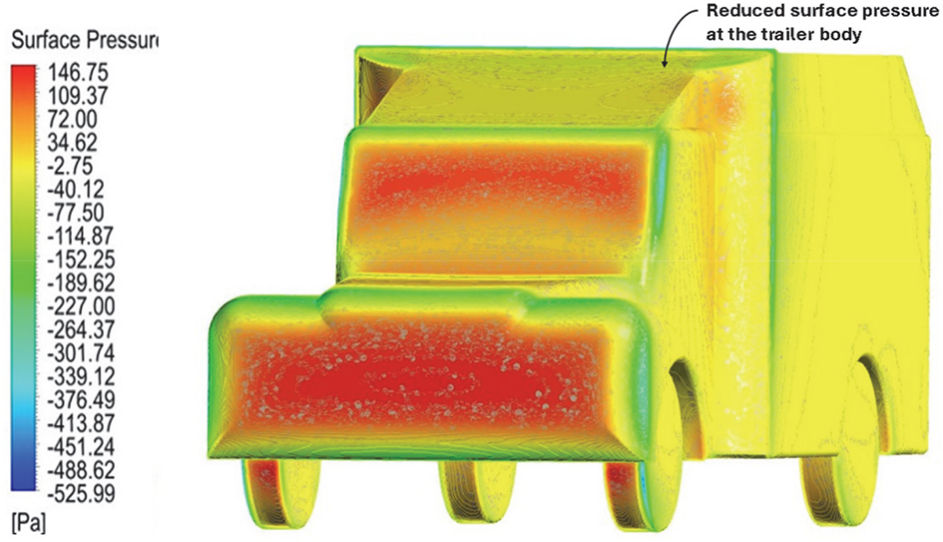

Table 19 shows the changes in different drag coefficient values. Effective design optimization results in a significant decrease in the drag of the truck geometry. An overall drag value of C _D_ = 0.45 for the optimised model is achieved for varying velocity conditions. The maximum drag reduction is achieved at 90 km/h. At higher speeds, the drag value was minimal for the optimized truck when compared to the baseline model.

**Table 19.** Comparison of baseline and optimized models.

Velocity (Kmph)	Baseline model	Optimised model	% Drag reduction
40	0.49	0.40	18.3
50	0.50	0.42	16.0
60	0.68	0.45	33.8
70	0.72	0.47	34.7
80	0.92	0.49	46.7
90	1.20	0.51	57.5

A significant drag reduction was obtained from the optimized model analysis. The flow modifications at the rear of the truck are observed owing to the effective slant angle of the square-back. The gap enclosure successfully eliminated flow recirculation in the truck-trailer gap region. This study successfully reduced the overall drag of the baseline truck using various design optimizations and effective turbulence models using a numerical method. Under high-speed conditions, the truck’s overall drag value was reduced in the case of the optimized model ( Figures 30 and 31)

**Figure 30.**
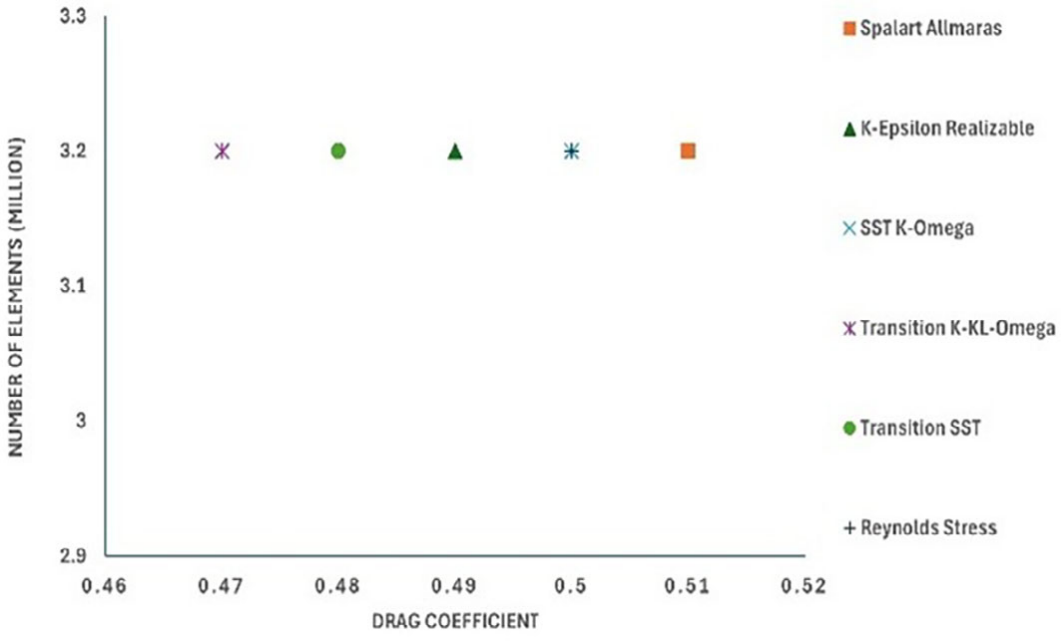
CRF model 3: Turbulence test.

**Figure 31.**
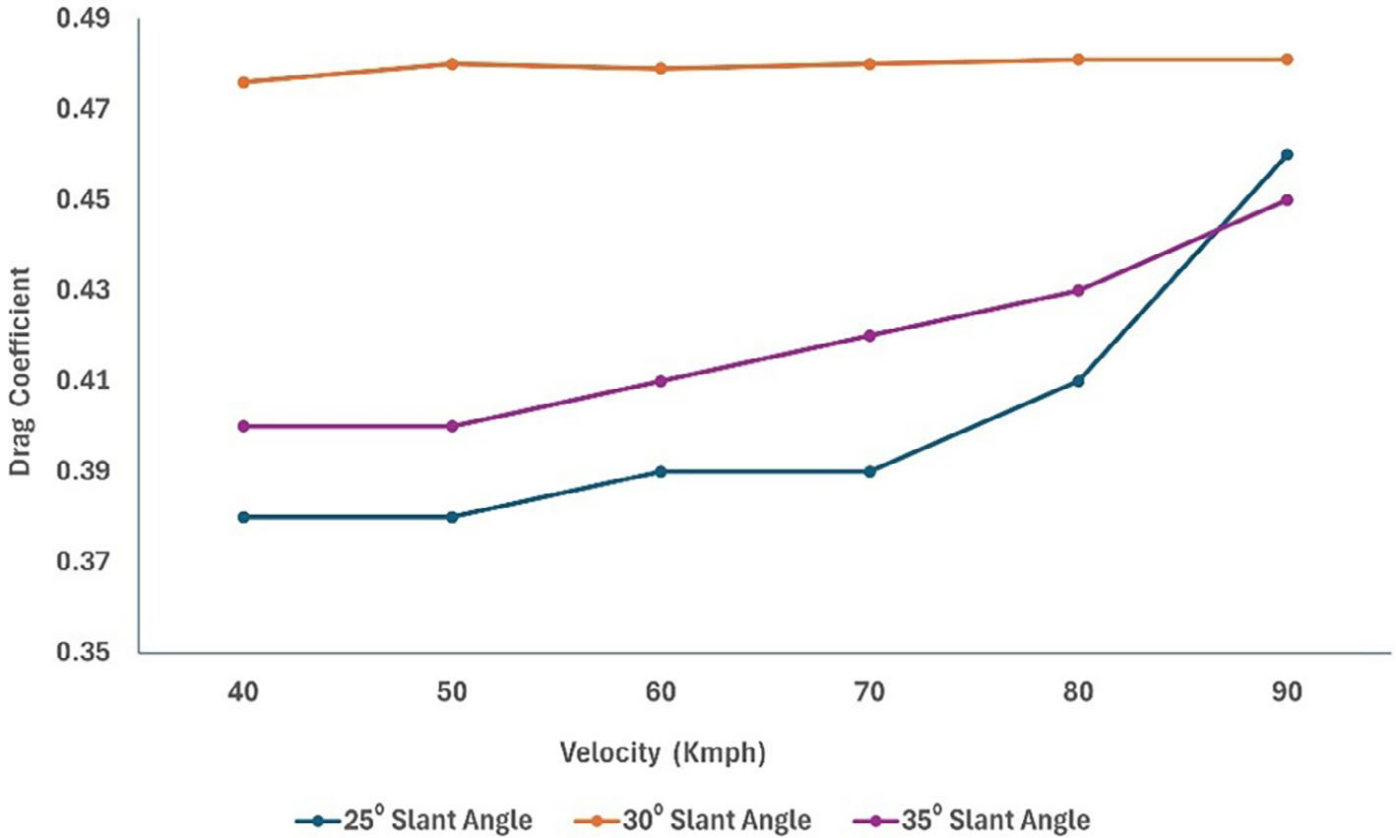
Varying velocity vs C _D_ for slant angle models.

**Figure 32.**
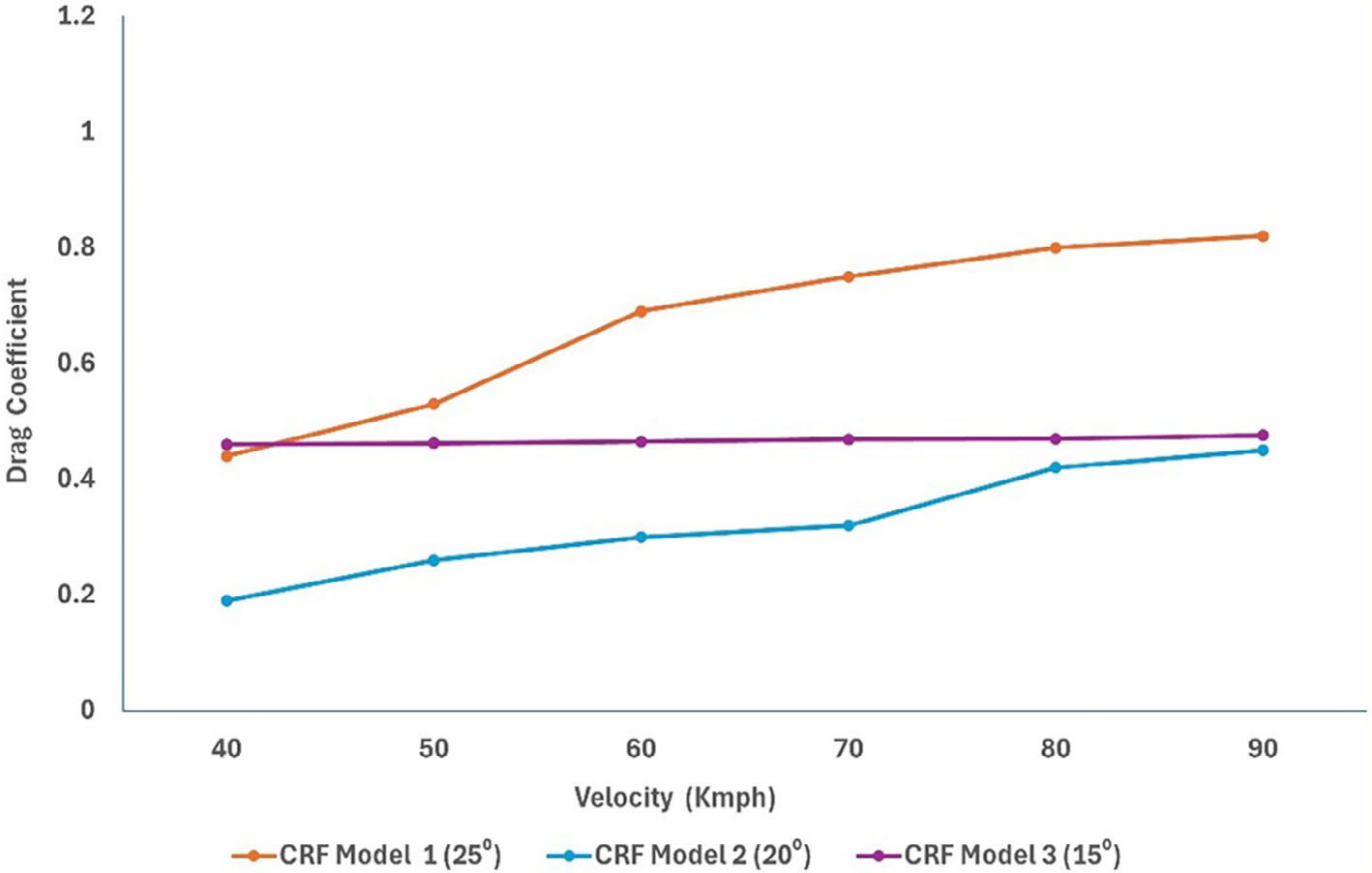
Varying velocity vs C _D_ for CRF models.

.

**Figure 33.**
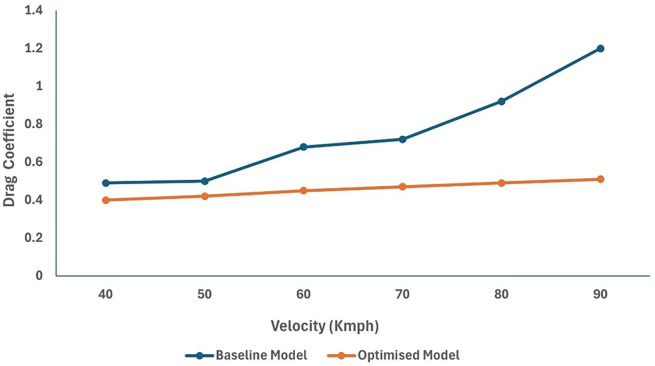
Comparison graph of C _D_ for baseline and optimised model.

The optimized model resulted in an effective 18% drag reduction when compared to the baseline model for an economy speed of 40 km/h. As shown in Figure 33, the overall drag coefficient value for the optimized model was much lower than that of the baseline model.

**Figure 34.**
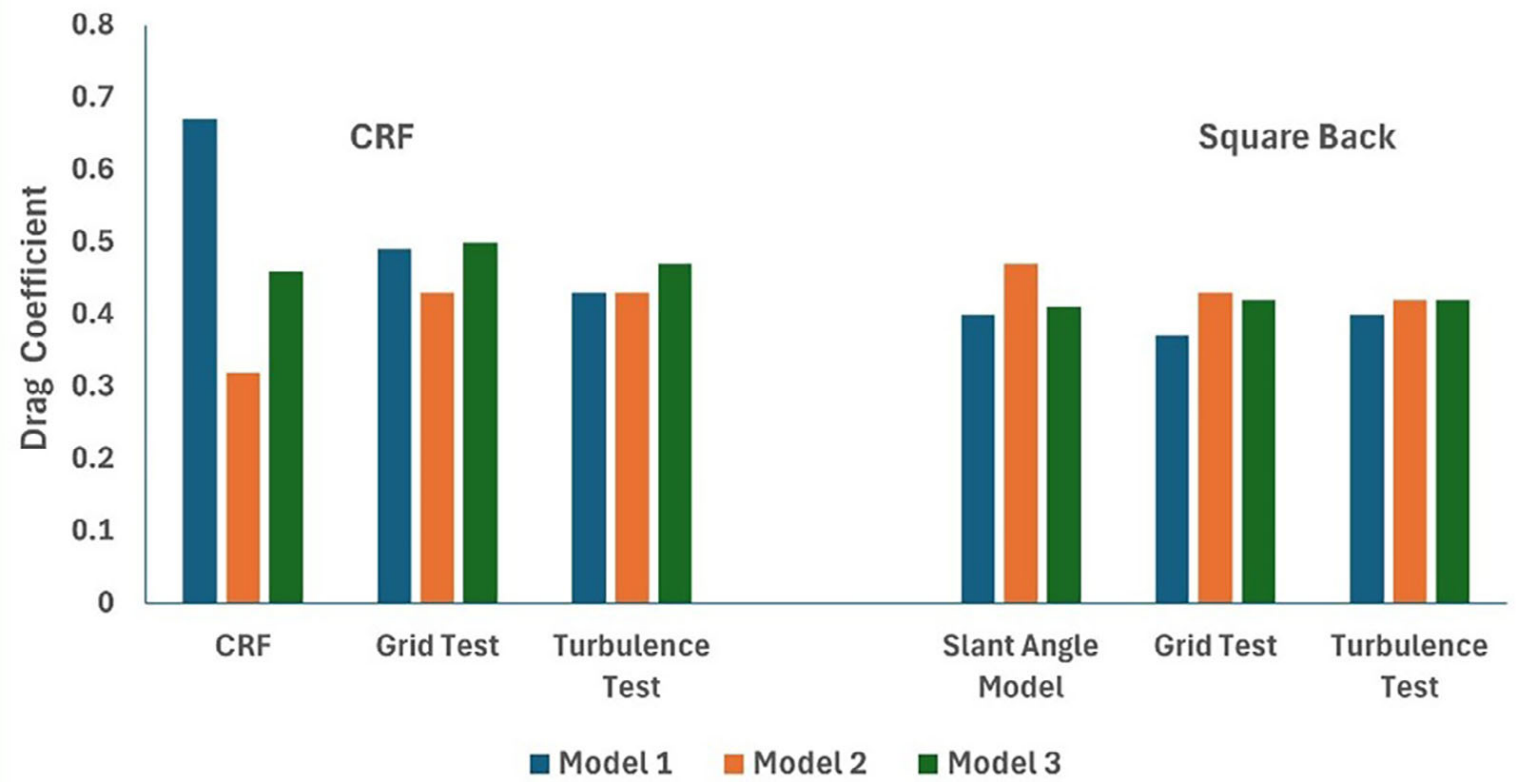
Drag values of various parameters for CRF and square back models.

In Figure 34, we can observe that the grid and turbulence tests have moderate drag values, and the final optimized model performs better than the baseline and reference paper models, indicating improvements in overall aerodynamics.

**Figure 35.**
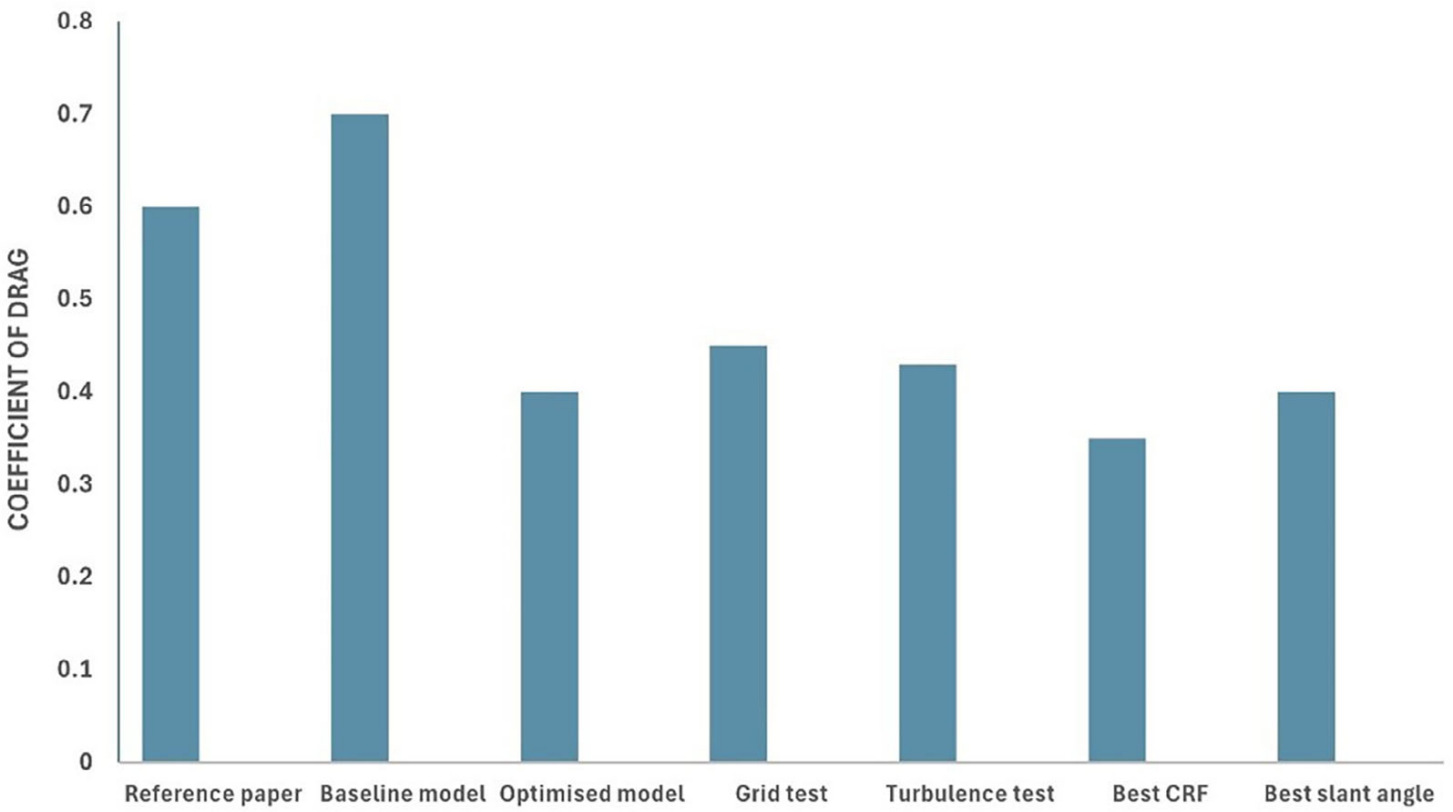
Best of the comparative drag coefficient values.

Figure 35 shows a comparison of the drag coefficients of the different models. The baseline model had the highest drag, while the optimized model, best CRF model, and best slant angle model showed significant reductions, indicating improved aerodynamic efficiency.

In Table 20, the C _D_ values of all models for different parameters are given. Lower drag coefficient values

**Table 20.** Drag coefficient values of various parameters.

	Drag Coefficient (C _D_)		
	Model 1	Model 2	Model 3
**CRF Model**	0.67	0.32	0.46
**Grid Test**	0.49	0.43	0.5
**Turbulence Test**	0.43	0.43	0.47

	Model 1	Model 2	Model 3
**Slant Angle Model**	0.4	0.47	0.41
**Grid Test**	0.37	0.43	0.42
**Turbulence Test**	0.4	0.42	0.42

.

## 5. Conclusions and future works

The objective of this study was to investigate the airflow behavior around a truck. In this study, a cab-roof fairing for effective drag reduction is proposed. A numerical analysis was performed for the baseline truck model, and the results obtained were compared and validated with reference papers. The least computational error for the baseline analysis was 2.8%. Passive drag reduction devices, such as cab roof fairing, proved to be an effective method for improving the overall aerodynamic efficiency of trucks by reducing drag.

The obtained drag coefficient value of the simulation was accurate and closer to the experimental reference value. Different turbulence models were tested, and the K-epsilon turbulence model, both standard and realizable, resulted in accurate results and a minimum computational error of 4%. The K-epsilon turbulence model proved to be very effective for free-flow conditions by providing a faster solution convergence.

Various CRF models and slant angle modifications were employed to optimize the truck. Two of the best models out of the nine design optimizations were chosen for the final simulation. Various grid tests were conducted to determine the optimal mesh for the simulations. The gap enclosures remarkably reduced the streamwise vorticity in the gap region between the truck cabin and the trailer. The wind deflector achieved optimal results by smoothly directing the airflow. The fairings delayed the flow separation on the side surfaces and blocked any inward flow into the gaps, thereby effectively reducing the drag. These modifications were effective in reducing the drag of the truck model.

The optimized model results showed a significant drag reduction at higher speeds. A drag reduction of 18 % %was achieved for the optimized model through effective CRF installation. The flow contours were analyzed and compared with the baseline model results. The optimized slant angle model results showed a reduced wake region size at the rear with an improved airflow structure around the truck cabin. The velocity streamline plot shows smooth flow lines above the optimized truck cabin.

Enhancing the aerodynamic features of the entire vehicle is essential. Various components, such as side skirts, underbody coverings, and gap extenders, are currently underutilized in commercial vehicles. Enhancements to the gap enclosure are required because a complete enclosure will obstruct the side air intake vent in the SCV and LCV. Hence, it is necessary to optimize the shape and geometry of aerodynamic devices to reduce drag and improve aerodynamic efficiency.

## Ethics and consent

Ethics and consent were not required.

## Data Availability

All underlying data are available in the article itself.
