# Supplementary material for: Aerodynamic Drag Reduction in Commercial Vehicle Using CFD-Based Design Optimisation
Source: F1000Res. 2025 Aug 26;14:821. [Version 1] doi: 10.12688/f1000research.163917.1 (PMC12789861; doi:10.12688/f1000research.163917.1)
Supplement: Peer review history

# Reviewer report: Reviewer response for version 1

**DOI:** 10.5256/f1000research.180338.r409653

**Published:** 2026-01-09

**Authors:** Ajith Raj

**Affiliations:** 1 Department of Aerospace Engineering, Karunya University, Coimbatore, Tamil Nadu, India

This article presents a CFD based investigation of aerodynamic drag reduction for a small commercial vehicle. The authors conduct a comprehensive numerical study using ANSYS Fluent to analyze baseline aerodynamic performance and apply multiple passive drag reduction strategies, including cab roof fairings (CRF), gap enclosures, and rear square-back slant modifications inspired by the Ahmed body. Grid independence studies, turbulence model comparisons, and velocity variation analyses are carried out to validate the baseline model against available experimental data. Several geometric configurations are evaluated iteratively, leading to an optimized truck configuration that demonstrates up to 18% drag reduction at economic speeds and significantly higher reductions at highway speeds.

 The study shows a result that passive aerodynamic devices, particularly optimized CRF and rear slant angles are effective in reducing drag and improving aerodynamic efficiency for commercial trucks.

Is the work clearly and accurately presented and does it cite the current literature?

Yes

If applicable, is the statistical analysis and its interpretation appropriate?

Yes

Are all the source data underlying the results available to ensure full reproducibility?

Yes

Is the study design appropriate and is the work technically sound?

Yes

Are the conclusions drawn adequately supported by the results?

Yes

Are sufficient details of methods and analysis provided to allow replication by others?

Yes

Reviewer Expertise:

Unmanned Aerial Vehicles, Composites, Electron Beam Welding, Dissimilar Metal Joints

I confirm that I have read this submission and believe that I have an appropriate level of expertise to confirm that it is of an acceptable scientific standard.

# Reviewer report: Reviewer response for version 1

**DOI:** 10.5256/f1000research.180338.r412239

**Published:** 2025-09-23

**Authors:** CİHAN BAYINDIRLI

**Affiliations:** 1 Nigde Omer Halisdemir University, Nigde, Turkey

The study was conducted using CFD to evaluate aerodynamic optimization. There is no shortage of CFD analysis. The aerodynamic study results need to be validated. The aerodynamic improvements achieved through passive flow control in this paper have not been experimentally validated.To achieve this, the author should support the study's results with research findings from the literature. I recommend reviewing several articles on passive flow control. I'd like to see the final version of the article after making any necessary additions.

 1- https://jesr.ub.ro/journal/article/view/348

 2-https://www.jafmonline.net/article_2232_b6f783237632a8e8408027493eb8b384.pdf

 3-https://dergipark.org.tr/en/pub/isibted/issue/87619/1515727

Is the work clearly and accurately presented and does it cite the current literature?

Yes

If applicable, is the statistical analysis and its interpretation appropriate?

Yes

Are all the source data underlying the results available to ensure full reproducibility?

Partly

Is the study design appropriate and is the work technically sound?

Yes

Are the conclusions drawn adequately supported by the results?

No

Are sufficient details of methods and analysis provided to allow replication by others?

No

Reviewer Expertise:

Aeroddynamic, CFD, Wind Tunnel, Passive Flow Control

I confirm that I have read this submission and believe that I have an appropriate level of expertise to confirm that it is of an acceptable scientific standard.
